# [Cu_2_(trz-ia)_2_]—An Ultramicroporous Cu_2_ Paddle Wheel Triazolyl Isophthalate MOF: A Comparative Study of Its Properties in Dihydrogen Adsorption and Isotopologue Separation

**DOI:** 10.1021/acs.inorgchem.4c05225

**Published:** 2025-03-05

**Authors:** Sibo Chetry, Prantik Sarkar, Volodymyr Bon, Muhammad Fernadi Lukman, Andreas Pöppl, Michael Hirscher, Stefan Kaskel, Harald Krautscheid

**Affiliations:** † Faculty of Chemistry and Mineralogy, Leipzig University, Johannisallee 29, 04103 Leipzig, Germany; ‡ Max Planck Institute for Intelligent Systems, Heisenbergstrasse 3, D-70569 Stuttgart, Germany; § Institute of Separation Science and Technology, Friedrich-Alexander-Universität Erlangen-Nürnberg (FAU), Erlangen 91058, Germany; ∥ Department of Inorganic Chemistry I, Faculty of Chemistry and Food Chemistry, Technische Universität Dresden, Bergstrasse 66, 01069 Dresden, Germany; ⊥ Felix-Bloch-Institute of Solid-State Physics, Faculty of Physics and Earth Sciences, Universität Leipzig, Linnéstrasse 5, Leipzig 04103, Germany; # Advanced Institute for Materials Research (WPI-AIMR), Tohoku University, Aoba-ku, Sendai 980-8577, Japan

## Abstract

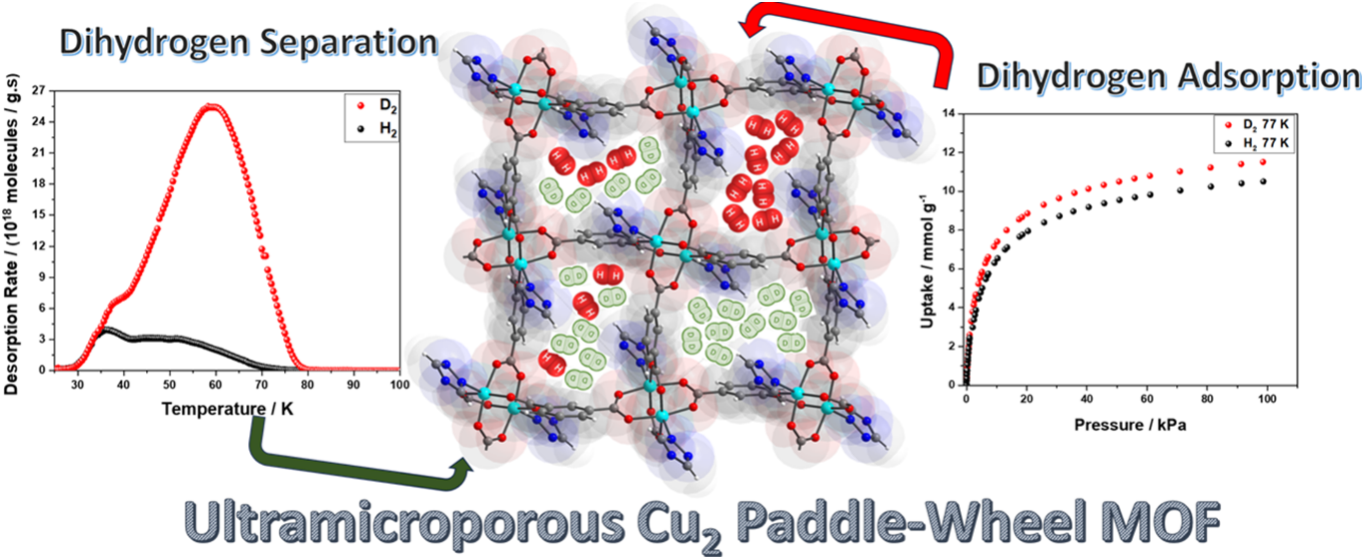

A Cu_2_ paddle wheel-based metal–organic framework, [Cu_2_(trz-ia)_2_] (trz-ia^2–^ = 5-(4*H*-1,2,4-triazol-4-yl) isophthalate), is investigated for hydrogen adsorption and hydrogen isotopologue separation. Its ultramicroporous structure with pore diameters ranging from 0.35 to 0.53 nm allows for strong interactions with dihydrogen molecules, resulting in steep H_2_ uptake and heat of adsorption *Q*_ads_ = 9.7 kJ mol^–1^. Notably, the hydrogen density inside the pores is 43.9 g L^–1^ at 77 K and 100 kPa. Thermal desorption spectroscopy (TDS) after exposure to a H_2_/D_2_ mixture indicates dihydrogen isotopologue separation with a selectivity of *S* = 6 at 30 K and a high uptake of D_2_. These findings are compared with numerous other metal–organic frameworks (MOFs) and related to their pore size.

## Abstract 2

A Cu_2_ paddle wheel-based metal−organic framework, [Cu_2_(trz-ia)_2_] (trz-ia^2−^ = 5-(4*H*-1,2,4-triazol-4-yl) isophthalate), is investigated for hydrogen adsorption and hydrogen isotopologue separation.

## Introduction

Hydrogen adsorption and isotope separation are at the forefront of the study due to the effective use of hydrogen as an energy carrier and development of renewable energy technologies. Hydrogen gas storage^1^ and separation of its isotopes, deuterium and tritium, both play a crucial role in various applications, such as molecular tracing,^2^ NMR spectroscopy,^3^ and nuclear reactors.^4,5^ D_2_ and T_2_ are potential fuels for nuclear fusion, offering hope for future energy solutions.^4,5^

Metal–organic frameworks (MOFs) are materials that are beneficial for gas storage and separation due to their large surface areas, high porosity, and tunability.^6^ Research into alternative energy sources is prompted by environmental problems brought on by the use of fossil fuels and the restricted continuity of existing renewable energy sources. The high energy density by weight of hydrogen plus the fact that its only combustion product is water make it a potential fuel.^7^ At room temperature, compressed hydrogen at 70 MPa has a density of 38 g L^–1^, while liquid hydrogen at 20 K and atmospheric pressure reaches a density of 70.8 g L^–1^.^1^ One effective method of storing hydrogen is through adsorption in porous materials; for optimal performance, these materials should have high surface areas and small pore sizes for high H_2_ affinity. However, it is often difficult to stumble upon materials with both of these characteristics. Generally, higher surface areas in porous materials result in higher hydrogen uptake, e.g., 500 m^2^ g^–1^ typically leads to 1 wt % of H_2_.^1,6,8^ Also, smaller pores (<0.7 nm) demonstrate higher hydrogen uptake in the low-pressure range as well as higher heat of adsorption.^9−12^ Because van der Waals forces between neighboring atoms overlap when there is a small pore with a high surface curvature, this leads to a stronger interaction.^10^ MOFs are adaptable porous materials that can be engineered for varying pore sizes, enhancing hydrogen adsorption. MOFs with ultramicropores (<0.7 nm)^13^ and coordinatively unsaturated metal sites (CUS) often show high affinity for H_2_, making them promising for hydrogen storage at room temperature.^14^

Worthwhile, the dihydrogen isotopologue D_2_ is particularly uncommon in nature. The deuterium isotope D makes up 0.0156% of all hydrogen atoms in the oceans (0.0312% by mass). It is difficult to separate D_2_ from its isotopologues, such as H_2,_ HD, T_2_, and HT, due to their striking parallels in characteristics. Traditional methods for separating dihydrogen isotopologues, like cryogenic distillation and the Girdler sulfide process,^15,16^ are costly, energy-intensive, inefficient, and have a limited capacity for separation.^16^ Hence, MOFs are considered an attractive platform for isotopologue separation due to their diverse structural configurations, pore sizes,^17,18^ adaptable functionality,^19^ and flexibility.^20,21^ Thermal desorption spectroscopy (TDS) enables the direct measurement of selectivity following exposure to an equimolar H_2_/D_2_ combination. Using this methodology on MOFs has demonstrated remarkable selectivity, as reported in the literature,^19,22−29^ it is higher than the one with traditional industrial setups (∼2.5).^15^ There are two mechanisms widely recognized in the literature controlling hydrogen isotopologue separation: Kinetic quantum sieving (KQS), where heavier isotopes diffuse at lower temperatures faster than lighter isotopes when the pore size matches the de Broglie wavelength of the gas molecule,^30^ and chemical affinity quantum sieving (CAQS), where the preference for heavier isotopologue adsorption due to differing adsorption enthalpies is caused by the isotopes’ different molecular masses and corresponding vibrations with lower frequencies.^25^ Both methods of hydrogen isotopologue separation are applicable to MOFs. The KQS mechanism necessitates low temperatures and severe constraints on pore sizes within 0.30–0.34 nm.^18,25^ Also recently, third-generation MOFs, which exhibit a breathing effect or gate-opening phenomenon were also utilized for the separation of hydrogen isotopologues at certain temperatures to enhance KQS and contribute to highly selective gas separation.^20,21,24,32^ The CAQS effect requires strong adsorption sites, e.g., CUS, which is demonstrated in Cu(I)-MFU-4l.^31^ The need to develop a MOF with both a narrow pore channel and CUS stems from the idea that this combination will increase the molecular interaction with the gas, resulting in increased dihydrogen adsorption and isotopologue separation.

For the MOF synthesis, application of linkers based on anionic carboxylate groups and neutral 1,2,4-triazole groups is well-known in the literature.^33^ The low overall charge, resistance to oxidation, broad coordination chemistry, and functionalization potential of these linkers are among their benefits.^33−35^ Ultramicropores with 0.32−0.49 nm pore size have been found in Cu- and Zn-based 1,2,4-triazolyl isophthalate MOFs accordingly.^34^ Effects on the flexibility and enhanced CO_2_ adsorption are observed upon functionalization of these linkers,^34,36,37^ and defect-engineered MOFs built upon these systems have shown a notable increase in the H_2_ adsorption affinity,^38^ underscoring these systems’ versatility and promise for use in gas sorption applications.^34−37^

In this work, we investigated a copper-based triazolyl isophthalate MOF _∞_^3^[Cu_2_(trz-ia)_2_] (trz-ia^2–^ = 5-(4*H*-1,2,4-triazol-4-yl) isophthalate) reported previously.^34^ Our research encompasses a thorough examination of both the pore size and coordinatively unsaturated sites inside this MOF, as well as its application for dihydrogen adsorption and isotopologue separation. We explored hydrogen adsorption at low temperatures and moderate pressures and then used thermal desorption spectroscopy (TDS) to evaluate dihydrogen isotopologue separation, revealing the material’s preference for D_2_ over H_2_.

## Experimental Section

All materials and preparation methods are detailed in Sections S1–S3. The remaining details of the characterization instruments and methods are provided in Sections S4–S13.

## Results and Discussion

### Characterization of [Cu_2_(trz-ia)_2_]

**Figure 1:**
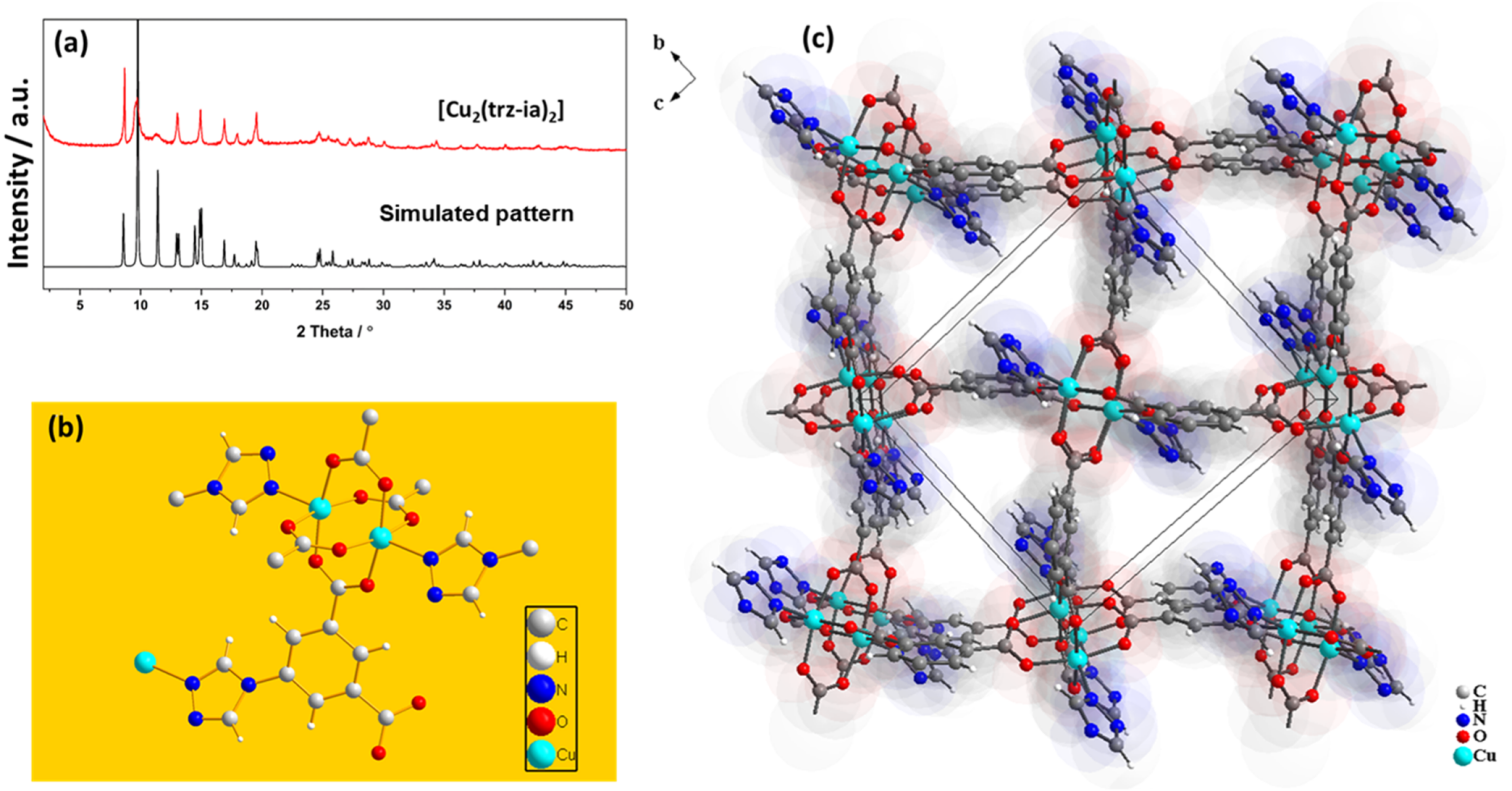
(a) PXRD pattern of as-synthesized [Cu_2_(trz-ia)_2_] measured at r.t. (λ = 154.060 pm) in comparison to the simulated PXRD pattern based on single-crystal data measured at 180 K,^34^ (b) fragment of the crystal structure with one paddle wheel unit, and (c) projection of the crystal structure of [Cu_2_(trz-ia)_2_] showing the pore channels along the crystallographic *a*-direction.

Phase-pure [Cu_2_(trz-ia)_2_] was synthesized in gram scale (Sections S2 and S3) using solvothermal and reflux techniques based on previously documented protocols.^34^ The powder X-ray diffraction (PXRD) pattern corresponds to the simulated pattern based on single-crystal data; however, some peaks are broadened, probably due to the particle size and solvent effects. The results of scanning electron microscopy (SEM) analysis are summarized in Section S12. The PXRD patterns (Figure 1a) do not change significantly upon solvent extraction and activation, confirming the structural rigidity of the [Cu_2_(trz-ia)_2_] network. Based on the single-crystal structure analysis, [Cu_2_(trz-ia)_2_] adopts **rtl** topology and contains the Cu_2_(O_2_CR)_4_ paddle wheel motif as a secondary structural unit.^34^ It crystallizes in the monoclinic space group *P*2_1_/*c* with two formula units [Cu_2_(trz-ia)_2_] per unit cell and contains narrow pore channels visible along the crystallographic *a*-axis, and the pore surface is formed by the aromatic rings of the ligand (Figure 1b,c and Section S4 and Figure S2). Eddaoudi et al. were the first to synthesize this MOF on a small scale, but scaling up was not achievable.^39^ Cheng et al. reported a network with the same composition but different structure and topology (**apo**).^40^ For checking the stability of [Cu_2_(trz-ia)_2_], we immersed samples in selected organic solvents and aqueous solutions at different pH values. According to PXRD results, the MOF is stable in the organic solvents; however, in aqueous media, a slow transformation to another network structure is observed (Section S5 and Figure S3).

[Cu_2_(trz-ia)_2_] was further characterized by gas physisorption. Whereas N_2_ shows no uptake (Section S10 and Figure S18), in contrast, the CO_2_ isotherms recorded at 195 and 298 K in the pressure range up to 100 kPa show significant adsorption with a type Ia isotherm with saturation loadings of 11 mmol g^–1^ at 195 K and 4.0 mmol g^–1^ at 298 K, indicating the highly ultramicroporous nature of this MOF (Section S10, Figures S19 and S20).

To investigate the pore system in more detail, the pore size distribution (PSD) calculated based on the crystal structure has been reported with pore diameters between 0.35 and 0.5 nm indicating ultramicroporosity.^34^ Similarly, the PSD determined from CO_2_ adsorption data at 298 K via the GCMC method also results in a bimodal pore system ranging from 0.34 to 0.53 nm pore diameter (Section S10 and Figure S21). A pore size around 0.30–0.35 nm is reported as the optimum pore diameter for hydrogen isotopologue separation governed by kinetic quantum sieving (KQS).^18,25^

**Figure 2:**
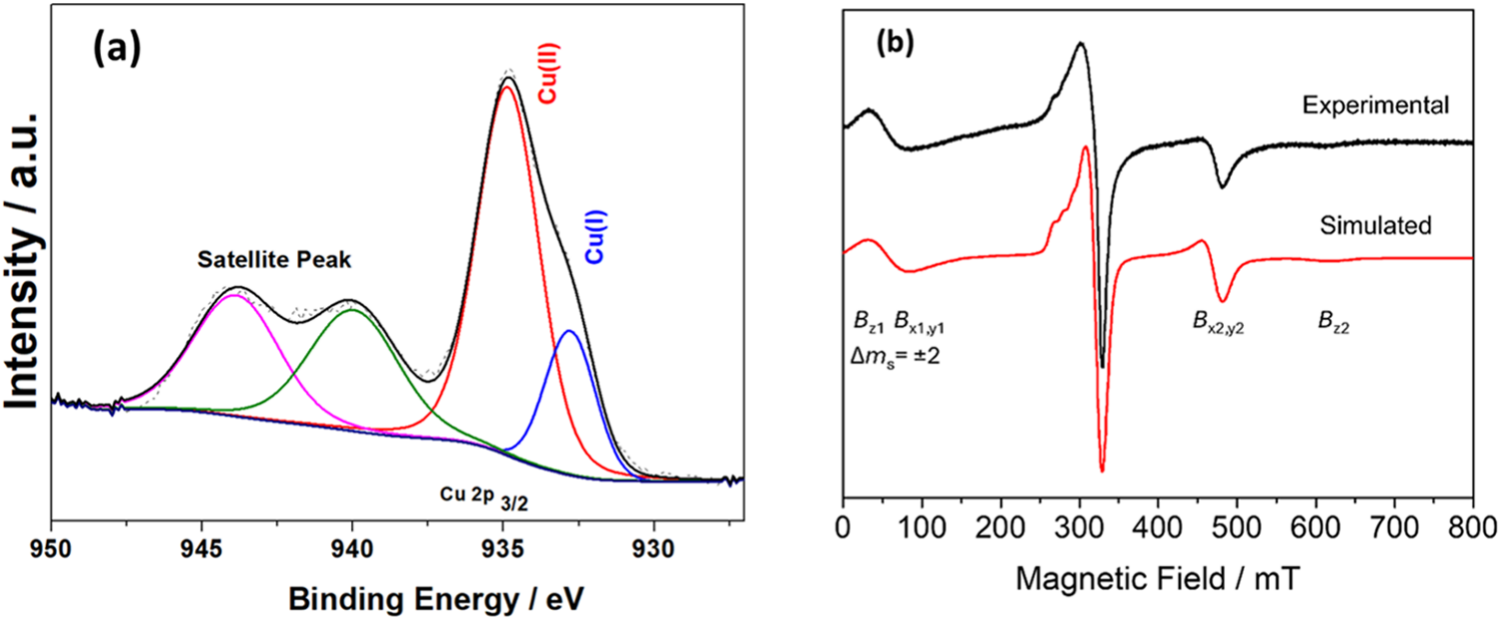
(a) Deconvoluted Cu 2p XPS spectra of [Cu_2_(trz-ia)_2_] and (b) *cw* X-band EPR spectrum of [Cu_2_(trz-ia)_2_] at 120 K (in black) and its spectral simulation (in red).

X-ray photoelectron spectroscopy (XPS) confirmed the presence of Cu^I^ in [Cu_2_(trz-ia)_2_] (Figure 2a). The survey XPS spectrum displays identifiable peaks corresponding to C 1s, O 1s, Cu 2p, and VB-XPS (survey and X-ray valence band spectra in Section S8 and Figures S7, S8b). The Cu 2p_3/2_ spectrum features two primary peaks at 934.6–935.1 and 932.8–933.2 eV, associated with Cu^II^ and Cu^I^, respectively.^38^ The X-ray valence band spectra of Cu also show two dominants at 4.6 eV and a small shoulder at 2 eV peaks, supporting the presence of Cu^I^ (Figure S8a). Further analysis of the Cu 2p_3/2_ signal in Figure 2a reveals distinct peaks at 935.1 and 933.2 eV, indicating the coexistence of Cu^II^ and Cu^I^ in [Cu_2_(trz-ia)_2_]. A similar result was obtained for a related methyl-substituted 1,2,4-triazolyl isophthalate Cu-based MOF in previously reported work.^38^ Through a peak fitting procedure carried out with CasaXPS,^41^ it was determined that approximately 19% of the copper species in [Cu_2_(trz-ia)_2_] exist in the Cu^I^ state, while the remaining 81% retain the Cu^II^ state. Previous studies indicated that XPS data alone might be inconclusive regarding the presence of defect sites due to the potential formation of Cu^I^ through various non defect-related mechanisms.^38^ To address this ambiguity and gain deeper insights into the defect sites, X-band continuous wave (*cw*) electron paramagnetic resonance (EPR) analysis was employed as a complementary technique.

X-band *cw* EPR spectroscopy was employed to study the local structure of the Cu_2_ dinuclear paddle wheel units within [Cu_2_(trz-ia)_2_]. Figure 2b indicates the EPR spectrum obtained at 120 K and its spectral simulation. It can be observed that at least two different species contributed to the EPR signal recorded at 120 K, Cu^II^–Cu^II^ paddle wheel units species (*S* = 1) and uncoupled mononuclear Cu^II^ species (*S* = 1/2).^38^ Further details on the spectral simulation for each spin system are given in Section S9 and Table S1. The signal related to *S* = 1 species stems from the interaction between two *S* = 1/2 spins from the interconnected Cu^II^–Cu^II^ dinuclear paddle wheel units, leading to an antiferromagnetic coupling with a ground-state level of *S* = 0 (diamagnetic singlet state) and an excited-state level of *S* = 1 (paramagnetic triplet state).^38,42,43^ This triplet state (*S* = 1) is thermally populated at higher temperatures, thus allowing the detection of the *S* = 1 signal by *cw* EPR above 60 K, as observed in the temperature dependence of *cw* X-band spectra (Section S9 and Figure S9). The spectral simulation for the spectra at 120 K yields an axially symmetric *g*-tensor (*g*_*xx*,*yy*_ = 2.080, *g*_zz_ = 2.355) and axially symmetric zero-field splitting (zfs) parameters *D* = 0.370 cm^–1^ and *E* = 0 cm^–1^ (Section S9 and Table S1). These values are slightly different from the values we obtained for the regular [Cu_2_(Me-trz-ia)_2_] MOF in our previous work,^38^ implying a slightly different local environment of the Cu^II^–Cu^II^ dinuclear paddle wheel units after the replacement of the methyl group with a proton in the organic linker. At the X-band frequency, there are four transitions to be observed under the condition of axially symmetric zfs (*D* ≠ 0 and *E* = 0): *B*_*x*1,*y*1_, *B*_*x*2,*y*2_, *B*_*z*1_, and *B*_*z*2_. However, *B*_*x*1,*y*1_ and *B*_*z*1_ transitions at *B* < 150 mT are superimposed with the signal of a forbidden EPR transition (Δ*m*_s_ = ± 2, where *m*_s_ is the magnetic spin quantum number) due to the comparable magnitude of *D* and microwave frequency. Our analysis for the uncoupled Cu^II^ signal (*B* = 240–380 mT) with partially resolved hyperfine interaction (hfi) coupling reveals an axially symmetric Cu^II^ species with the estimated *g*_*zz*_ = 2.355(5) and hyperfine coupling *A*_*zz*_ = 400(20) MHz. The origin of uncoupled Cu^II^ species can be related to extra-framework Cu^II^ impurities or Cu^II^ ions in defective paddle wheel units, which are not coupled into pairs within the paddle wheel units in the MOF structure.^42^ The ratio of such uncoupled Cu^II^ species (*N*_M_) relative to the Cu^II^–Cu^II^ dinuclear paddle wheel units (*N*_PW_) is estimated to 15% (Section S9.1).

### H_2_ and D_2_ Gas Adsorption Properties of [Cu_2_(trz-ia)_2_]

**Figure 3:**
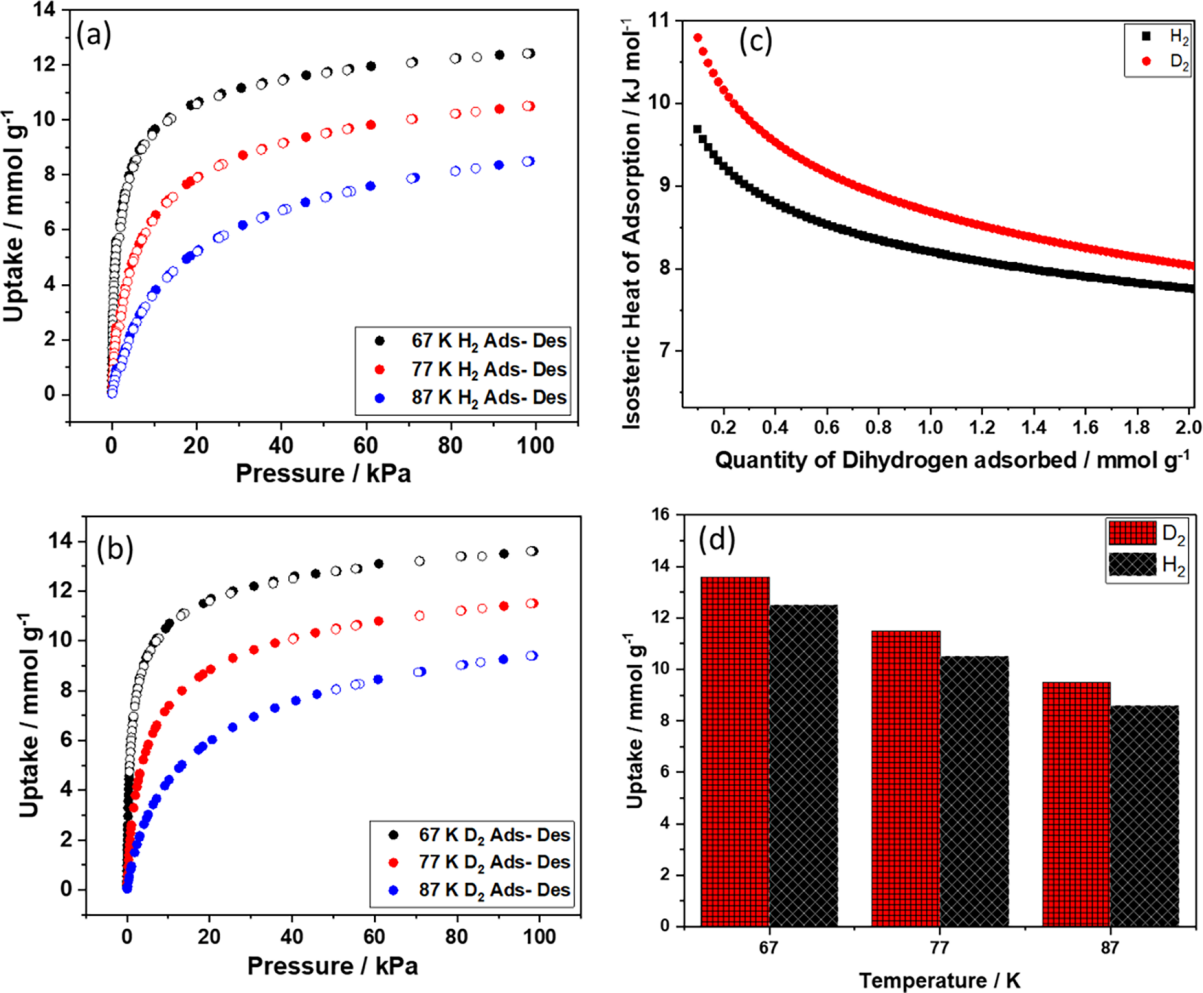
Low-pressure H_2_ (a) and D_2_ (b) adsorption–desorption isotherms on [Cu_2_(trz-ia)_2_] at 77 K, closed symbols correspond to adsorption, open symbols correspond to desorption, (c) heat of adsorption of H_2_ and D_2_ on [Cu_2_(trz-ia)_2_], and (d) comparison of H_2_ and D_2_ adsorption data at 67, 77, and 87 K.

To evaluate the [Cu_2_(trz-ia)_2_] material’s dihydrogen adsorption behavior, the pure gas H_2_ and D_2_ adsorption isotherms were acquired, as shown in Figure 3a,b. At 77 K and 100 kPa, [Cu_2_(trz-ia)_2_] demonstrates 10.5 and 11.5 mmol g^–1^ uptake of H_2_ and D_2_, respectively (Figure 3d). This noticeable difference of 1.0 mmol g^–1^ in the adsorption capacity between D_2_ and H_2_ underscores the superior adsorption performance of D_2_ as compared to H_2_ across the entire range of pressure conditions. This phenomenon can be attributed primarily to distinctive nuclear quantum effects differing for D_2_ and H_2_,^25^ which alter the adsorption behavior in a notable manner. As depicted in Figure 3c, the heat of adsorption (*Q*_ads_) of H_2_ and D_2_ at [Cu_2_(trz-ia)_2_] is determined using the Clausius–Clapeyron equation in the temperature range of our experiment, 67–87 K; the specific methodology and calculations are described in Section S10 and Figures S10, S11. The heat of adsorption for hydrogen on [Cu_2_(trz-ia)_2_] of *Q*_ads_ = 9.7 kJ mol^–1^ for H_2_ and 10.9 kJ mol^–1^ for D_2_ at a low coverage decreases to 7.5 kJ mol^–1^ at a high coverage (Figures 3c and S12). In general, this value is a bit lower than the literature indicates for the heat of H_2_ adsorption for MOFs with CUS sites between 10 and 32 kJ mol^–1^.^6^ The observed reduction in *Q*_ads_ with increased loading can be attributed to the initial filling of ultramicropores (0.34 nm), which offer stronger interactions due to their size and geometry. As these smaller pores fill, the interaction energy decreases, leading to a more uniform heat of adsorption at higher coverages as larger pores become occupied. Detailed information on *Q*_ads_ is provided in Section S10 and Figure S12; this highlights the efficiency of ultramicropores in enhancing sorption due to the overlap of interaction potentials from the pore walls.^9−11^

To further elucidate the adsorption sites within [Cu_2_(trz-ia)_2_], advanced thermal desorption spectroscopy (TDS) data were acquired for pure H_2_ and D_2_. Activated MOF samples were exposed to pure gas at ambient temperature and 1 kPa for 10 min. Subsequently, the sample was cooled to 20 K, followed by evacuation to remove nonadsorbed molecules and heated at a rate of 0.1 K s^–1^. As depicted in Section S11 and Figure S22, the TDS profile for [Cu_2_(trz-ia)_2_] exhibits a single desorption maximum for both pure H_2_ (60.9 K) and pure D_2_ (63.3 K). The observed isotopic shift indicates a higher adsorption enthalpy for D_2_ compared to H_2_. TDS analysis did not reveal any distinct desorption maxima at higher temperatures (above 70–100 K) that would be characteristic of such CUS as observed in Cu-MFU-4l and MOF-74.^19,31^ Furthermore, to support this finding, Zeo++^44^ calculations were performed, which are a versatile tool designed for the analysis of crystalline porous materials. It allows for geometry-based evaluation of the void space, structural topology, modifications, or assembly of structures, and it supports both single-crystal structure analysis and high-throughput analysis of large databases. Zeo++ calculations were performed to calculate CUS sites (open metal sites) in this MOF. However, according to geometric considerations in Zeo++, the metal ions are not exposed in a way that would justify considering them as open metal sites. Consistent with the correlation between the pore size and desorption temperature reported by Krkljus et al. and Panella et al.,^12,45^ the ultramicroporous nature of [Cu_2_(trz-ia)_2_] (pore radii: 0.34–0.53 nm) is likely responsible for the observed relatively high desorption temperatures, whereas a higher desorption temperature indicates a stronger interaction potential. As the pore size decreases to <0.7 nm, the interaction potential increases.^10,12^

Also, studies by Dincǎ et al.^46^ on ultramicroporous MOFs reported steep gas uptake at low pressures, accompanied by *Q*_ads_ of 10.1 kJ mol^–1^. These findings highlight the significant role of ultramicropores in enhancing gas adsorption at a low pressure as well as higher *Q*_ads_. Given the structural similarities, it is proposed that the steep gas uptake of dihydrogen at a low pressure along with higher *Q*_ads_ and desorption maxima centered at 60 (63.3) K for H_2_ (D_2_) are related to ultramicroporosity. The desorption maxima are observed at a lower temperature than expected for CUS; the TDS curves for many MOFs showed that the CUS lead to a higher desorption temperature.^12,45^ This implies that practically no CUS in [Cu_2_(trz-ia)_2_] is accessible and, as a result, does not contribute to the overall adsorption behavior of the MOF.

### Comparison of [Cu_2_(trz-ia)_2_] with Other MOFs with Varying Pore Sizes, Hydrogen Uptake, and *Q*_ads_ at a Low Coverage

**Figure 4:**
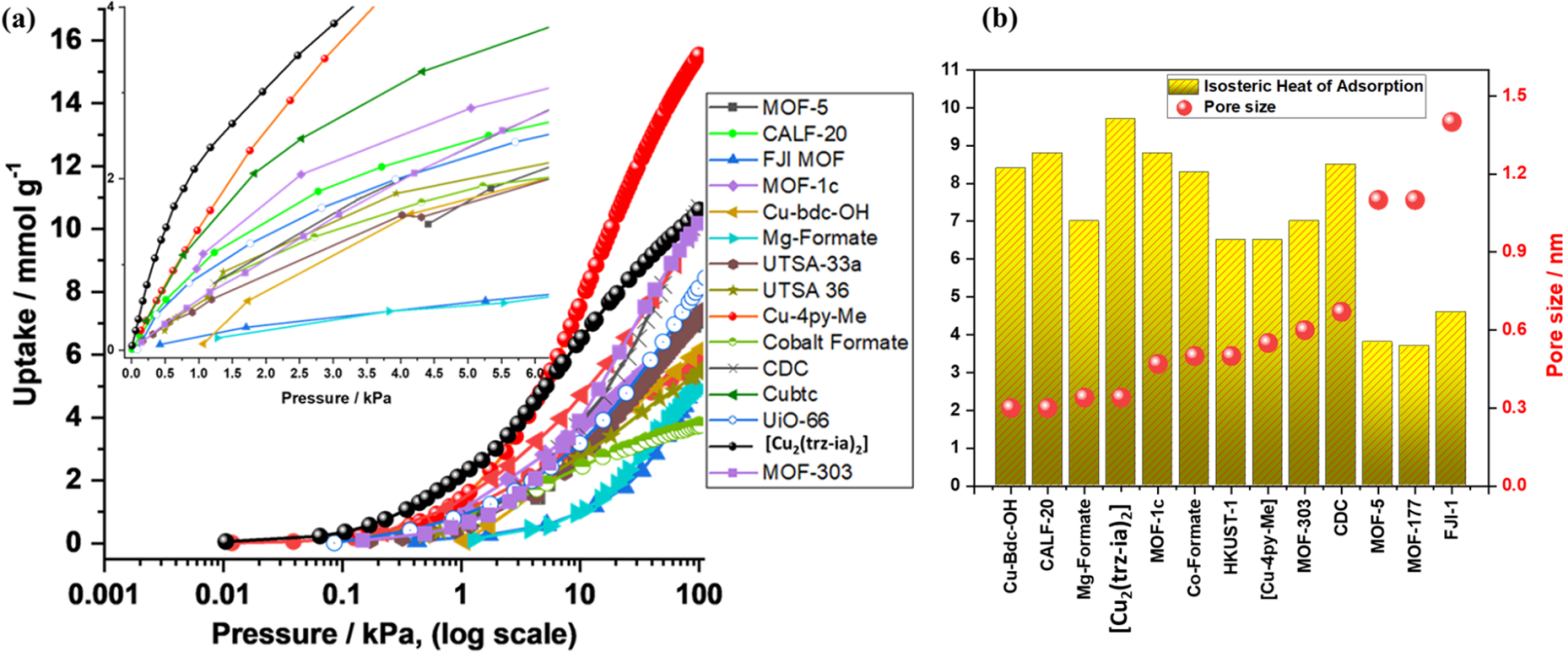
(a) Comparison of hydrogen uptake by various MOFs with varying pore sizes used for H_2_ gas adsorption at 77 K and up to 100 kPa and (b) pore sizes and heat of H_2_ adsorption of various MOFs.

For comparison of the hydrogen adsorption properties of [Cu_2_(trz-ia)_2_] with various well-known MOFs with different pore sizes, we synthesized and characterized CALF-20,^17^ MOF-303,^28^ HKUST-1,^47^ [Cu-4py-Me],^48^ and UiO-66,^49^ examining their H_2_ gas adsorption and *Q*_ads_ (detailed in Section S10 and Figures S10–S17). Our analysis includes H_2_ adsorption isotherms recorded at 77 K and measured up to 100 kPa, as well as *Q*_ads_ measured at the lowest coverage, which corresponds to the smallest pores. To complement our experimental data, we obtained gas adsorption information from the NIST database and gathered pore diameters and *Q*_ads_ for additional MOFs from literature sources (referenced in Section S10 and Table S2).^8,9,28,32,40,47−56^ These results are compiled in Figure 4a, which highlights the sharp and steep H_2_ gas uptake by [Cu_2_(trz-ia)_2_] compared to other MOFs, which display a less pronounced uptake behavior at a low pressure. This indicates a much stronger interaction between the H_2_ molecule and the pore walls of [Cu_2_(trz-ia)_2_]. The pore sizes (smallest pore in the case of bimodal pore distribution) and enthalpies *Q*_ads_ are compared in Figure 4b, showcasing a correlation between *Q*_ads_ and the pore size. *Q*_ads_ is within 6–10 kJ mol^–1^ in the case of all ultramicroporous MOFs, while MOFs with larger pore sizes above 0.7 nm demonstrate lower *Q*_ads_ (<5 kJ mol^–1^).

Additionally, we looked into the volumetric uptake of H_2_ on [Cu_2_(trz-ia)_2_] at 77 K and 100 kPa, which is 23 g L^–1^. The density of hydrogen inside the pore can be calculated using the pore volume and results in 43.9 g L^–1^ at 77 K and 100 kPa, which is denser than compressed H_2_ at 70 MPa with 38 g L^–1^ and more than half the density of liquid hydrogen at its boiling temperature 70 g L^–1^^1,8^ (calculation given in Section S10.3).

### Isotope Separation Efficiency

**Figure 5:**
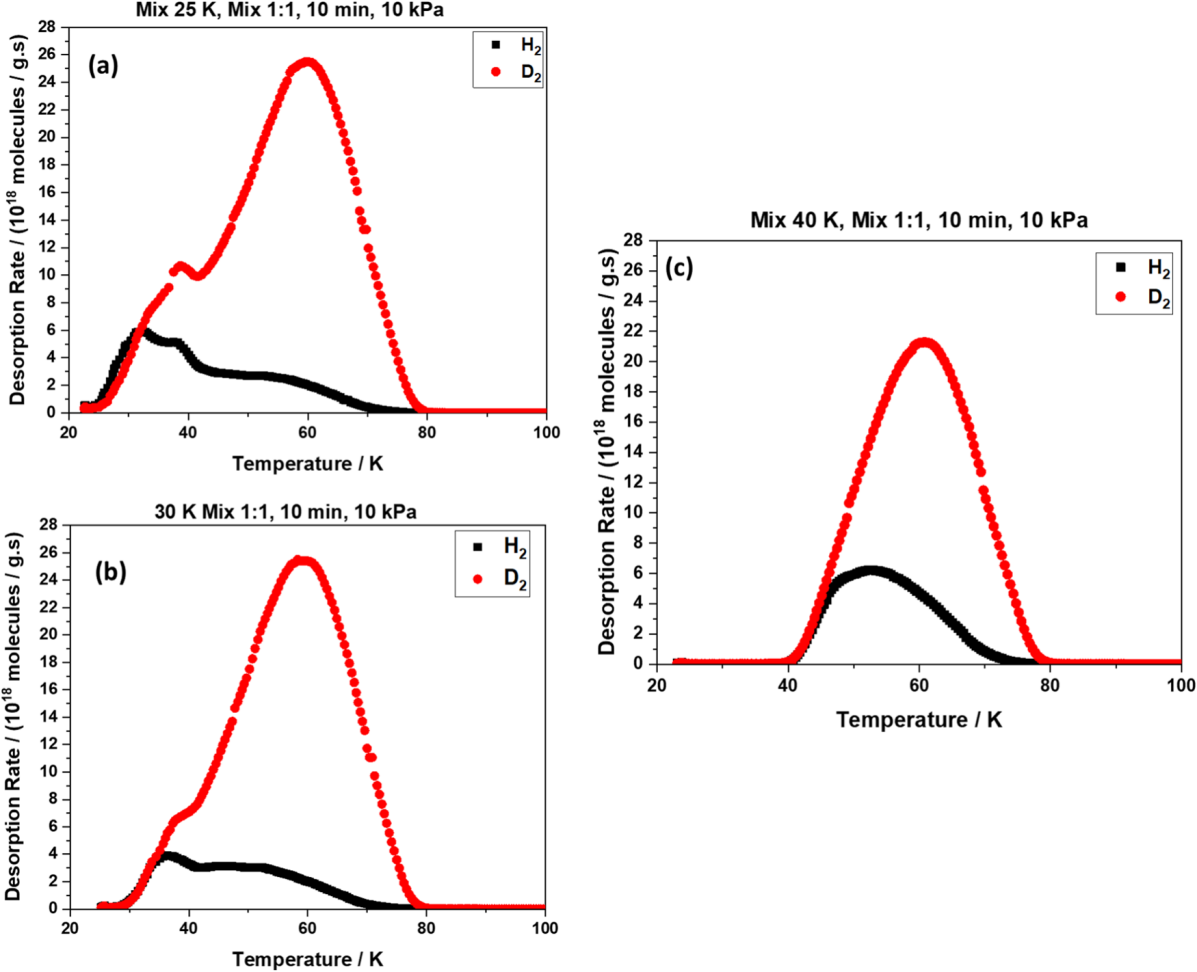
Thermal desorption spectroscopy (TDS) graphs for 1:1 D_2_/H_2_ gas mixtures subjected to [Cu_2_(trz-ia)_2_] at various exposure temperatures and 10 kPa exposure pressure: (a) 25 K, (b) 30, and (c) 40 K. H_2_ and D_2_ are represented by black and red symbols, respectively.

[Cu_2_(trz-ia)_2_] exhibits a bimodal pore structure with pore sizes of 0.34–0.53 nm (Section S10 and Figure S21), whereas 0.30–0.35 nm has previously been identified as optimal for dihydrogen isotopologue separation via kinetic quantum sieving.^25,57^ To evaluate the MOF’s separation performance, TDS experiments were conducted on a 1:1 D_2_/H_2_ mixture (10 kPa exposure pressure) adsorbed on [Cu_2_(trz-ia)_2_] at exposure temperatures of 25–40 K (Figure 5).

Prior to each measurement, the system was thoroughly evacuated to eliminate nonadsorbed gas molecules. For 25 and 30 K exposure temperature, TDS data show two desorption peaks: a weaker peak at 34–36 K (H_2_) and 38–39 K (D_2_) assigned to dihydrogen weakly adsorbed at the surface of the MOF and a stronger peak at 52–54 K (H_2_) and 61 K (D_2_) for hydrogen confined in ultramicropores. After exposure at 40 K, only one desorption maximum is observed, centered at 54 K for H_2_ and at 61 K for D_2_. Selective adsorption of D_2_ over H_2_ is evident in the TDS spectra, where the relative area under the desorption peaks indicates the isotopic composition of the desorbed gas.^25^ For a 1:1 equimolar mixture of D_2_ and H_2_ (10 kPa) at an exposure temperature of 30 K, [Cu_2_(trz-ia)_2_] exhibits a significantly higher D_2_ adsorption capacity (10.5 mmol g^–1^) compared to many other MOFs (see Table 1). Under these conditions, the selectivity, *S* = 6, defined as the ratio of D_2_ to H_2_ desorption, is lower than that of several other reported MOFs.^27^ The variation of selectivity with exposure temperature, demonstrated to be decreasing from 25 to 40 K (Section S11 and Figure S23), is in agreement with the expected trend for the KQS effect. However, for the CAQS effect, the opposite trend is expected. Thus, the separation in [Cu_2_(trz-ia)_2_] is attributed to the KQS effect of the ultramicropores.

**Table 1:** Comparison of the D_2_/H_2_ Separation Performance of Various Porous Materials

MOFs	*P_e_*_xposure_ [kPa]	*T_e_*_xposure_ [K]	*S*_D_2_/H_2__1:1 mixture	*D*_2_ uptake [mmol/g]	refs
DUT-8(Ni)	80.0	23.3	11.6	9.4	(20)
MOF-303	100.0	25	21	18.6	(28)
MIL-53(Al)	1.0	25	2.83	22.1	(21)
Ni-MOF-74	1.0	77	19	4.0	(19)
oIFP-3	1.0	30	2.41	1.6	(18)
IFP-1	1.0	30	1.69	4.3	(18)
CoFA	100.0	25	26	7.0	(32)
Py@COF-1	2.6	22	9.7	0.5	(57)
Co(pyz)[M(CN)_4_], M = Pd,Ni,Pt	1.0	25	21.7 (Pd), 17.8 (Ni), 16.1 (Pt)	10.5 (Pd),11.2 (Ni), 9.7 (Pt)	(26)
Cocryst1	1.0	30	7.7	4.72	(29)
CC3	1.0	30	1.7	3.6	(29)
6FT-RCC3	1.0	30	2.2	2.8	(29)
6ET-RCC3	1.0	30	3.9	0.3	(29)
[Cu_2_(trz-ia)_2_]	10.0	25	5	11.4	this work
[Cu_2_(trz-ia)_2_]	10.0	30	6	10.5	this work
[Cu_2_(trz-ia)_2_]	10.0	40	3.6	7.5	this work

## Conclusions

The construction and comprehensive characterization of the ultramicroporous MOF [Cu_2_(trz-ia)_2_] demonstrate that it has a high potential for dihydrogen adsorption and isotopologue separation. Due to ultramicroporosity (pore diameters below 0.7 nm) of the material, which leads to pronounced overlap of the van der Waals interactions of the neighboring atoms at a low pressure, the interactions are stronger than in MOFs with bigger pore sizes. While XPS and EPR studies confirmed the presence of structural defects, such as Cu^I^ and uncoupled Cu^II^ species, TDS measurements revealed no high-temperature desorption maximum (>70 K), indicating that these defects do not influence the material’s adsorption behavior. This is further supported by Zeo++ calculations, which suggest that the adsorption behavior is primarily governed by confinement within the ultramicropores. The stronger adsorption enthalpy of D_2_ compared to H_2_ extracted from the isotopic shift of TDS spectra and the temperature-dependent decrease in selectivity corroborate that kinetic quantum sieving (KQS) is the dominant separation pathway. At a low temperature, exposure to an isotopologue mixture reveals higher D_2_ uptake compared to many other MOFs, together with a D_2_ over H_2_ selectivity of ∼6.

Overall, this study underscores the importance of ultramicroporosity in governing the adsorption and separation properties in MOFs. The findings provide valuable insights for the design of new ultramicroporous materials for hydrogen storage and isotope separation applications.

## Supporting Information Available

Experimental information; characterization data from synthesized materials; single-crystal data; PXRD patterns; thermal analysis data; FTIR, XPS, EPR spectra; gas adsorption data; TDS; SEM; and elemental analysis results (PDF)
