# Supplementary material for: [Cu2(trz-ia)2]—An Ultramicroporous Cu2 Paddle Wheel Triazolyl Isophthalate MOF: A Comparative Study of Its Properties in Dihydrogen Adsorption and Isotopologue Separation
Source: Inorg Chem. 2025 Mar 5;64(10):5077–85. doi: 10.1021/acs.inorgchem.4c05225 (PMC11920950; doi:10.1021/acs.inorgchem.4c05225)
Supplement: Supplementary file 1 — ic4c05225_si_001.pdf [file ic4c05225_si_001.pdf]

Supporting Information

[Cu_2(trz-ia)$_2$] – an Ultramicroporous Cu_2 Paddle Wheel Triazolyl Isophthalate MOF: A Comparative Study of its Properties in Dihydrogen Adsorption and Isotopologue Separation

Sibo Chetry[a], Prantik Sarkar[b,c], Volodymyr Bon[d], Muhammad Fernadi Lukman[e], Andreas Pöppl[e], Michael Hirscher[b, f], Stefan Kaskel[d], Harald Krautscheid[a*]

[a] Faculty of Chemistry and Mineralogy, Leipzig University, Johannisallee 29, 04103 Leipzig, Germany

[b] Max Planck Institute for Intelligent Systems, Heisenbergstrasse 3, D-70569 Stuttgart,Germany

[c] Institute of Separation Science and Technology, Friedrich-Alexander-Universität Erlangen-Nürnberg (FAU), Erlangen 91058, Germany

[d] Department of Inorganic Chemistry I, Faculty of Chemistry and Food Chemistry, Technische Universität Dresden, Bergstrasse 66, 01069 Dresden, Germany

[e] Felix-Bloch-Institute of Solid-State Physics, Faculty of Physics and Earth Sciences, Universität Leipzig, Linnéstrasse 5, Leipzig 04103, Germany

[f] Advanced Institute for Materials Research (WPI-AIMR), Tohoku University, Aoba-ku, Sendai, 980-8577, Japan

*Corresponding Author: Prof. Dr. Harald Krautscheid

krautscheid@rz.uni-leipzig.de

Contents

S1 Chemicals 4
S2 Ligand Synthesis 5
S3 MOF Synthesis 5
S4 Single Crystal Structure of [Cu_2(trz-ia)$_2$] 6
S5 Powder X-ray diffraction 8
S6 FT-Infrared spectra 12
S7 Thermal Gravimetric Analysis 13
S8 X-ray photoelectron Spectroscopy and EPR Spectroscopy 14
S9. Electron Paramagnetic Resonance 16
S9.1. Calculation of mononuclear defect species in [Cu_2(trz-ia)$_2$] 17
S10 Gas Adsorption 20
S10.1. Pore Size Distribution 20
S10.2. Calculation of the number of H_2 molecules adsorbed per unit cell of MOF 21
S10.3.Calculation of volumetric hydrogen uptake and hydrogen density 22
S10.4. Comparison of hydrogen adsorption data obtained from NIST Database. 24
S10.5. Isosteric Heat of Adsorption 24
S10.6. N_2 and CO_2 Sorption Studies 29
S11 Thermal Desorption Spectroscopy (TDS) 32
S12 Scanning Electron Microscopy (SEM) 34
S13 Elemental analysis 36
S14. Reference 37

List of Tables and Figures

Table S1. Simulation parameters for Cu^{II} related species in the [Cu_2(trz-ia)$_2$] MOF. 18
Table S2. Data for several MOF adsorption isotherms, acquired at 77 K and 100 kPa pressure. 22
Table S3. Elemental analysis of [Cu_2(trz-ia)$_2$] 36

Figure S1. Unit cell of the crystal structure of [Cu_2(trz-ia)$_2$] showing the coordination of the ligand trz-ia^{2-} and the paddle wheel units. 6
Figure S2. Crystal structure of [Cu_2(trz-ia)$_2$] showing the pore channels along the crystallographic a-axis. The sizes of the black arrows are 3.5 Å × 6.0 Å. 7
Figure S3. (a) PXRD patterns of [Cu_2(trz-ia)$_2$] under various conditions, compared with the simulated pattern derived from single-crystal data measured at 180 K. (b) PXRD patterns of [Cu_2(trz-ia)$_2$] in different solvents. (c) Water stability assessment of [Cu_2(trz-ia)$_2$] over time. (d) pH stability evaluation of [Cu_2(trz-ia)$_2$] across a range of pH values. 8
Figure S4. PXRD patterns of HKUST-1, MOF-303, CALF-20, [Cu-4py-Me], UiO-66 in comparison with simulated patterns based on single crystal data. 11
Figure S5. FT-IR spectra of [Cu_2(trz-ia)$_2$] and dimethylammonium-3-carboxy-5-(4H-1,2,4-triazol-4-yl) benzoate (pure ligand). 12
Figure S6. The TG-DTA-MS analysis showcases the evaporation of guest molecules (below 160 °C) and the decomposition of the framework (above 260 °C), as evidenced by the detected MS signals for $(H_2O)^+$ (m/z=18), $(MeO)^+$ (m/z=31), and $(CO_2)^+$ (m/z=44). 13
Figure S7. XPS survey scan spectra of [Cu_2(trz-ia)$_2$] 14
Figure S8. (a) X-ray Valence Band Cu spectra (b) Cu LMM Auger spectra of [Cu_2(trz-ia)$_2$] 15
Figure S9. (a) Deconvolution of cw X-band EPR spectra of [Cu_2(trz-ia)$_2$] at 120 K according to the spectral simulation of S = 1 and S = 1/2 species. (b) temperature-dependent cw EPR spectra and (c) Temperature dependence of the normalized EPR intensity of the S = 1 species using the $B_{x2,y2}$ signal. Solid curve is the best fit to the Bleaney–Bowers equation. 19
Figure S10. Langmuir-Freundlich fit for D_2 and H_2 isotherms of [Cu_2(trz-ia)$_2$] at 67, 77 and 87 K. 25
Figure S11. Isosteric plot of ln (p) against 1/T for different loadings n (in mmol g^{-1}) of [Cu_2(trz-ia)$_2$] with D_2 and H_2 gas at 67, 77 and 87 K. 25
Figure S12. Langmuir-Freundlich fits and Clausius-Clapeyron equation were used to calculate Q_{ads} for D_2 (red) and H_2 (black) adsorption isotherms on [Cu_2(trz-ia)$_2$] samples at 67, 77 and 87 K, respectively. 26
Figure S13. H_2 gas adsorption isotherm, Langmuir-Freundlich fit, and heat of adsorption for HKUST-1. Symbols – experimental data, lines – fit based on Langmuir-Freundlich model. 27
Figure S14. H_2 gas adsorption isotherm, Langmuir-Freundlich fit, and heat of adsorption for [Cu-4py-Me]. Symbols – experimental data, lines – fit based on Langmuir-Freundlich model. 27
Figure S15. H_2 gas adsorption isotherm, Langmuir-Freundlich fit, and heat of adsorption for MOF-303. Symbols – experimental data, lines – fit based on Langmuir-Freundlich model. 28
Figure S16. H_2 gas adsorption isotherm, Langmuir-Freundlich fit, and heat of adsorption for UiO-66. Symbols – experimental data, lines – fit based on Langmuir-Freundlich model. 28
Figure S17. H_2 gas adsorption isotherm, Langmuir-Freundlich fit, and heat of adsorption for CALF-20. Symbols – experimental data, lines – fit based on Langmuir-Freundlich model. 29
Figure S18. N_2 adsorption-desorption isotherms of [Cu_2(trz-ia)$_2$] recorded at 77 K up to 100 kPa. 29
Figure S19. CO_2 adsorption-desorption isotherms of [Cu_2(trz-ia)$_2$] recorded at 195 K up to 100 kPa. 30
Figure S20. CO_2 adsorption-desorption isotherm of [Cu_2(trz-ia)$_2$] recorded at 298 K up to 100 kPa. 30
Figure S21. PSD of [Cu_2(trz-ia)$_2$] calculated using GCMC method from CO_2 isotherm recorded at 298 K up to 100 kPa. 31
Figure S22. D_2 (red) and H_2 (black) TDS curves for [Cu_2(trz-ia)$_2$] after room temperature exposure to 1.0 kPa of pure H_2 and D_2 gas and then cooling to 20 K. 33
Figure S23. Comparison of D_2 uptake (black, left axis) and selectivity (red, right axis) for D_2 over H_2 as a function of temperature in the studied [Cu_2(trz-ia)$_2$]. 33
Figure S24. SEM images of [Cu_2(trz-ia)$_2$] 35

S1 Chemicals

Chemical	Formula	Vendor	Purity
Ethyl acetate (EtOAc)	$C_4H_8O_2$	VWR HiPerSolv	99.99%
Hydrazine monohydrate	$N_2H_4 \cdot H_2O$	Acros Organics	100 % (64 % Hydrazine)
Ethanol (EtOH)	C_2H_5OH	VWR HPLC Grade	99.99%
Methanol (MeOH)	CH_3OH	VWR HPLC Grade	99.99%
Sodium carbonate	Na_2CO_3	Fluka Analytical	-
5-Amino-1,3-isophthalic acid	$C_8H_7NO_4$	Acros Organics	99.95%
Copper (II)-chloride dihydrate	$CuCl_2 \cdot 2H_2O$	Merck	99.95%
Dimethylformamide (DMF)	C_3H_7NO	VWR HPLC Grade	99.99%
Thionyl chloride	$SOCl_2$	Acros Organics	99.95%
Xylene	$(CH_3)_2C_6H_4$	Acros Organics	98%

S2 Ligand Synthesis

N,N-Dimethyl formamide azine was obtained according to the procedure developed by Bartlett and Humphrey.[1] Dimethylammonium-3-carboxy-5-(4H-1,2,4-triazol-4-yl) benzoate was synthesized from 5-aminobenzoic acid and N,N-dimethyl formamide azine according to Lässig et al.[2]

S3 MOF Synthesis

Solvothermal synthesis of ${}_{\infty}^{3}[Cu_2(H_2\text{-trz-ia})_2]$, abbreviated as $[Cu_2(\text{trz-ia})_2]$ (trz-ia^{2-} = 5-(4H-1,2,4-triazol-4-yl)isophthalate): Similar to the procedure reported by Kobalz et al.,[3] a stainless-steel autoclave with Teflon insert was loaded with dimethylammonium-3-carboxy-5-(4H-1,2,4-triazol-4-yl) benzoate (5 mmol), Copper (II)-chloride dihydrate (5 mmol) and 10 ml DMF: EtOH (1:1 v/v), sealed, and the reaction mixture was heated within 1 h up to 120 °C. After maintaining the temperature at the same level for 5 h, the autoclave was cooled to room temperature within 60 h. The solvent mixture employed for synthesis was also used to wash the microcrystalline product. The MOF product was subject to MeOH exchange over the course of seven days. For further characterisation and measurements, the solvent was removed and the sample was activated in dynamic vacuum overnight.

Some MOFs were explored for hydrogen adsorption to compare with $[Cu_2(\text{trz-ia})_2]$: HKUST-1[4], UiO-66[5], MOF-303[6], [Cu-4-py-Me][7] and CALF-20[8] were synthesized according to the reported literature methods.

S4 Single Crystal Structure of [Cu_2(trz-ia)$_2$]

The results of the single crystal X-ray diffraction analysis of [Cu_2(trz-ia)$_2$] were already reported.[3] The structure diagrams are visualized using Diamond[9] software.

Formula unit: $C_{20}H_{10}Cu_2N_6O_8$

Formula weight: 589.42 g mol^{-1}

Space-group: $P2_1/c$; Z = 2

Unit cell at 180 K: a = 10.968(2), b = 12.2031(14), c = 14.366(2) Å, β = 110.304(13) °

Unit cell volume: 1803.3(5) Å^3

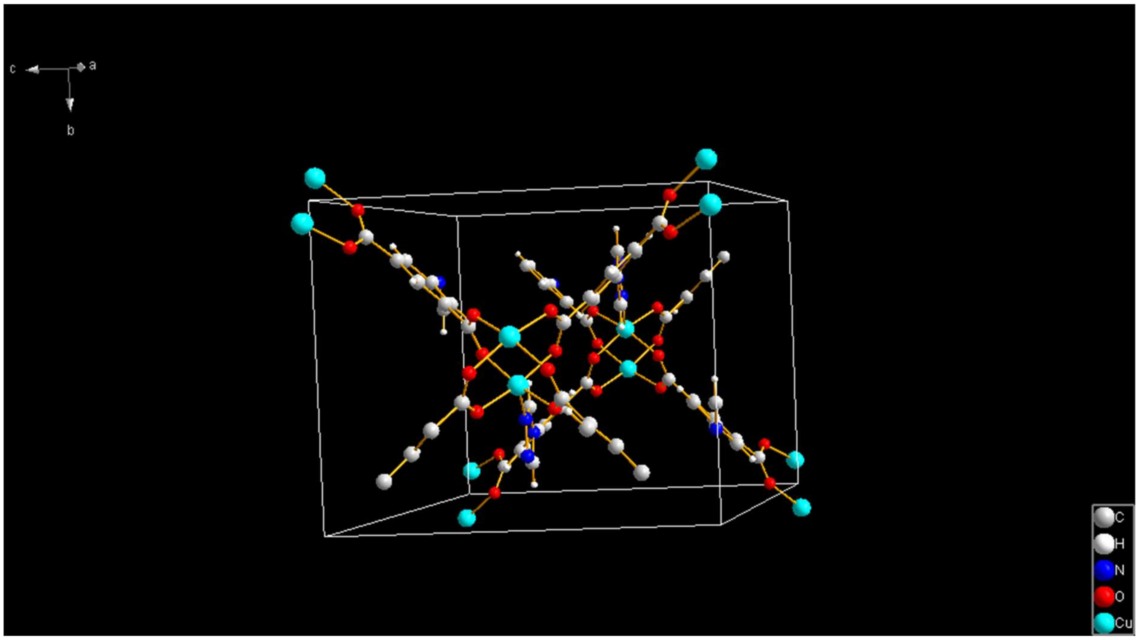

Figure S1. Unit cell of the crystal structure of [Cu_2(trz-ia)$_2$] showing the coordination of the ligand trz-ia^{2-} and the paddle wheel units.

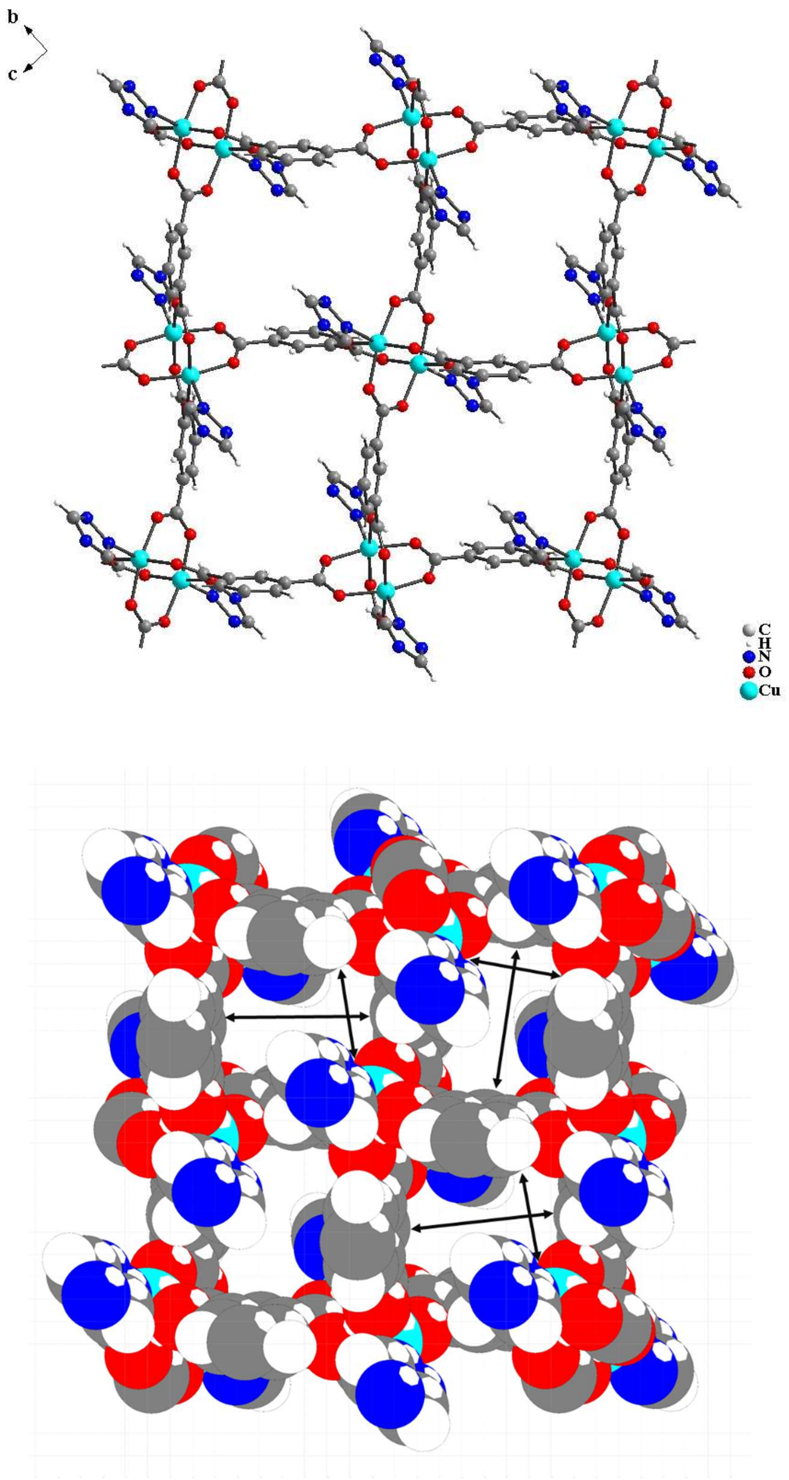

Figure S2. Crystal structure of [Cu_2(trz-ia)$_2$] showing the pore channels along the crystallographic a-axis. The sizes of the black arrows are 3.5 Å × 6.0 Å.

S5 Powder X-ray diffraction

Powder X-ray diffraction (PXRD) patterns were obtained at r.t. using a powder diffractometer STADI-P (Stoe & Cie GmbH) operating in transmission mode (Debye Scherrer mode) with Cu-$K_{\alpha 1}$ radiation (λ = 1.54060 Å). The samples for these measurements were prepared in glass capillaries (Hilgenberg, outer diameters 0.3 mm to 0.7 mm).

Compared to the simulated PXRD pattern, the measured reflections (r.t.) are slightly shifted to lower diffraction angles because the simulated pattern is based on single crystal data measured at low temperature (180 K)[3].

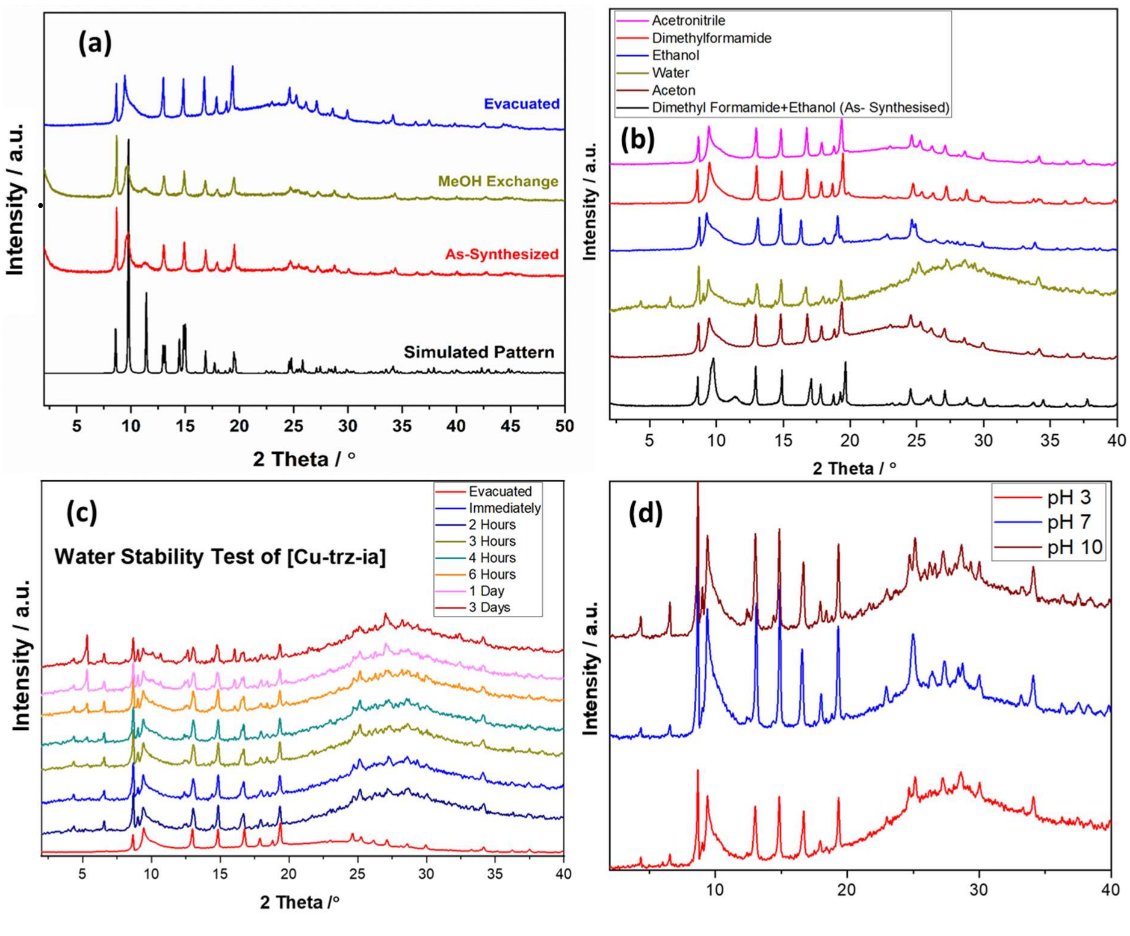

Figure S3. (a) PXRD patterns of $[Cu_2(trz\text{-}ia)_2]$ under various conditions, compared with the simulated pattern derived from single-crystal data measured at 180 K. (b) PXRD patterns of $[Cu_2(trz\text{-}ia)_2]$ in different solvents. (c) Water stability assessment of $[Cu_2(trz\text{-}ia)_2]$ over time. (d) pH stability evaluation of $[Cu_2(trz\text{-}ia)_2]$ across a range of pH values. The reflections below $2\theta=10°$ appearing in presence of water are due to transformation of $[Cu_2(trz\text{-}ia)_2]$ to a MOF with composition $[\{Cu_3(\mu_3\text{-}OH)(Anion)\}_4\{Cu_2(H_2O)_2\}_3(trz\text{-}ia)_{12}]$.[10]

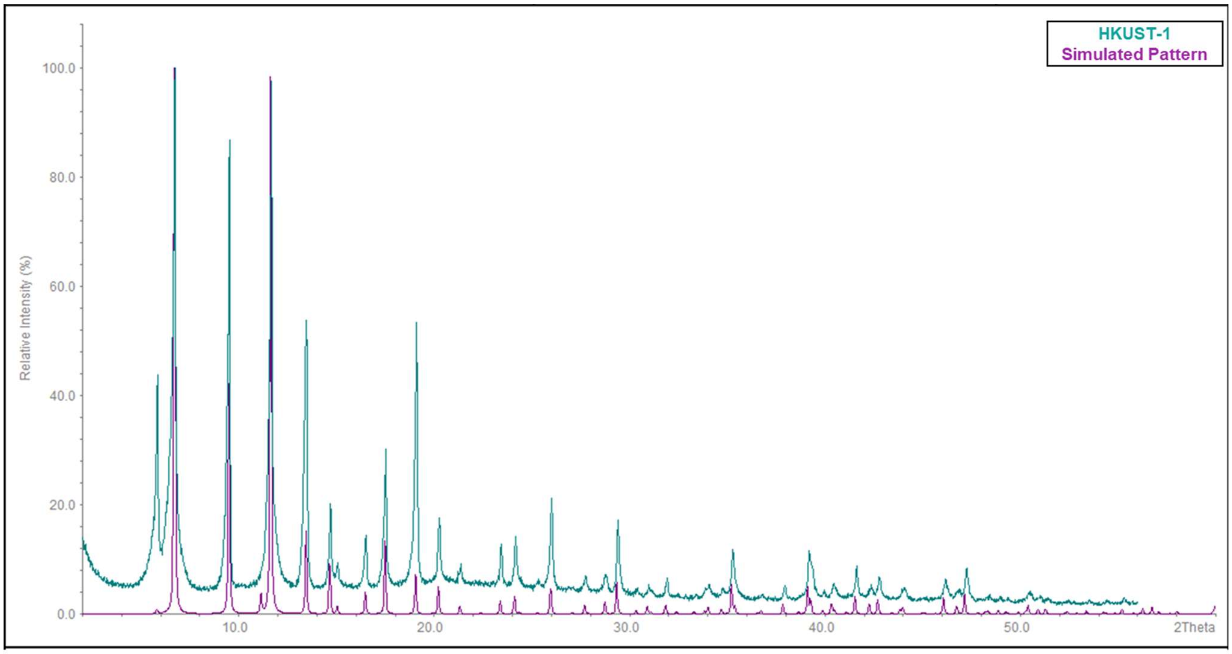

2 theta / °

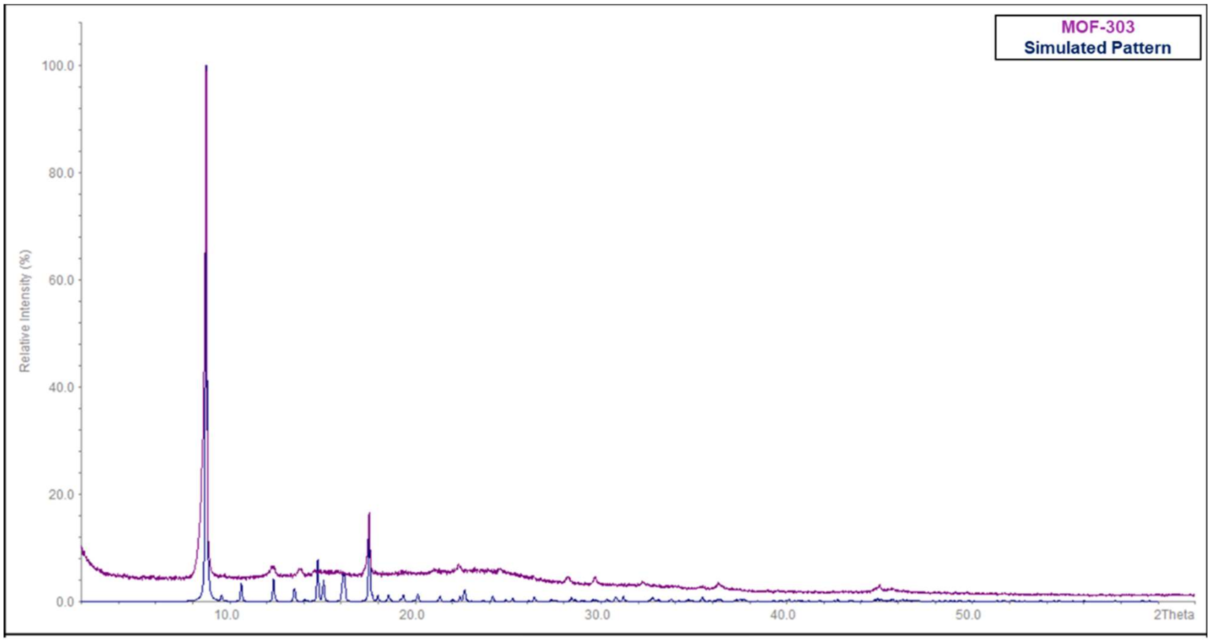

2 theta / °

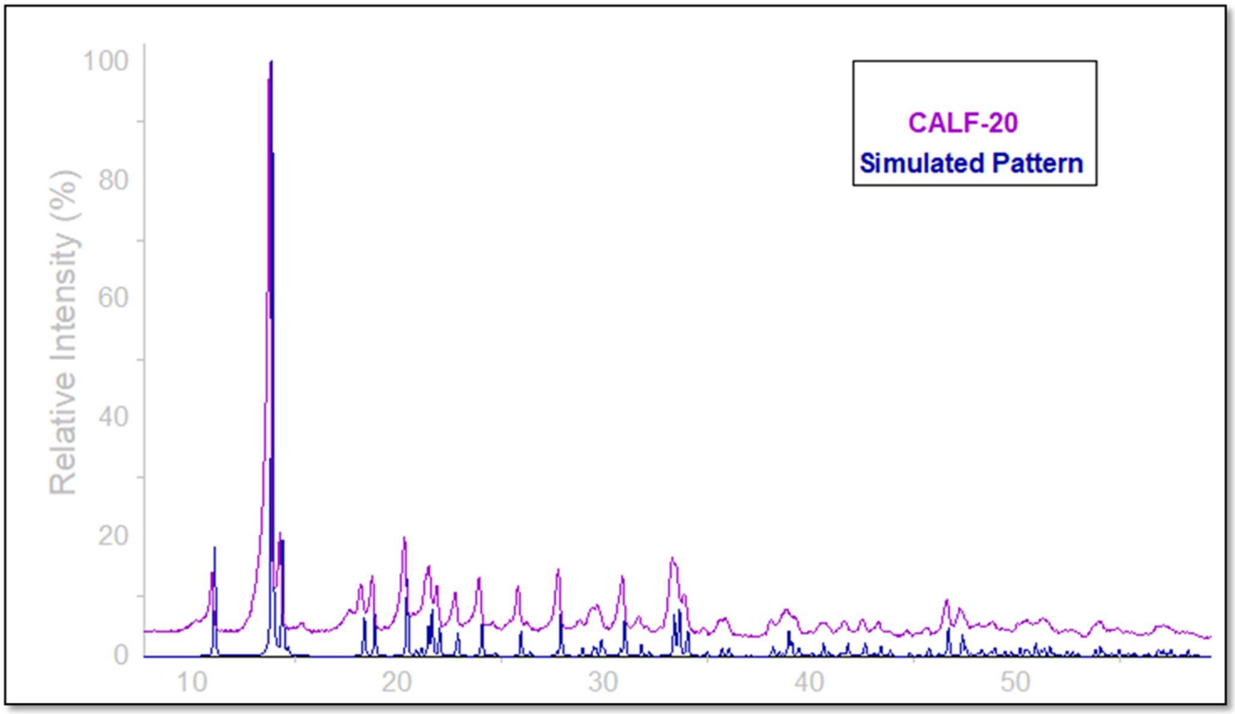

2 theta / °

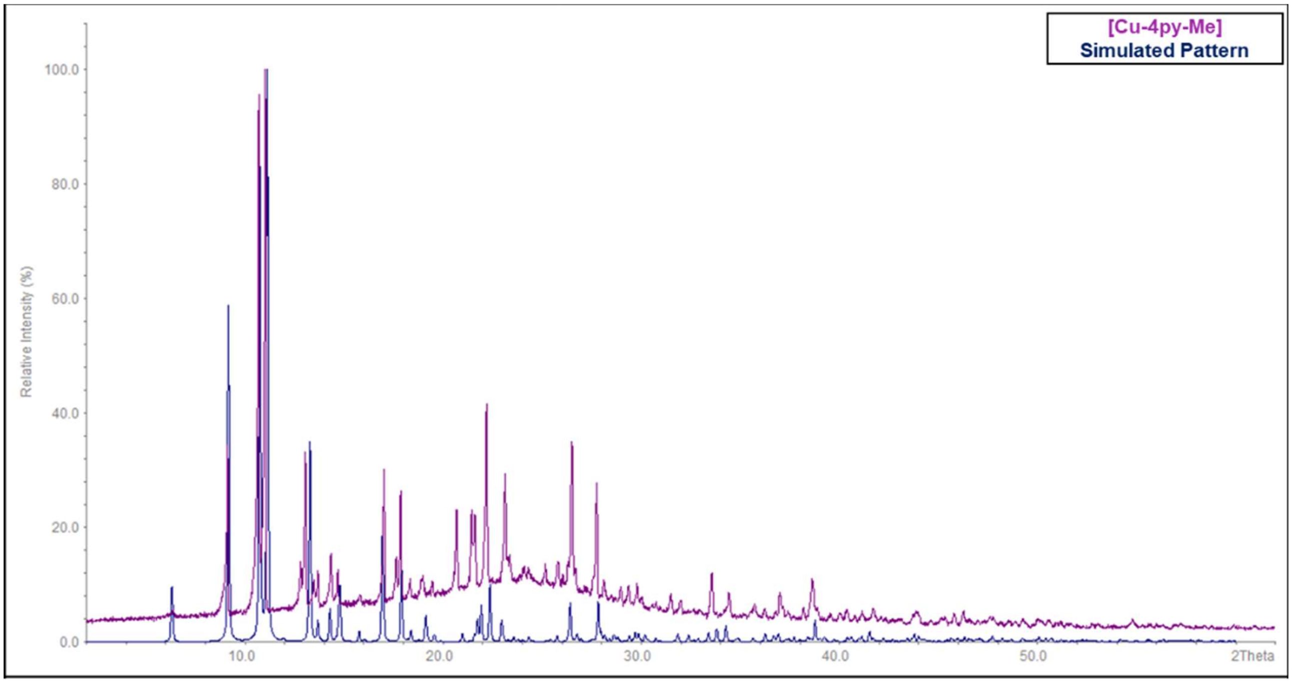

2 theta / °

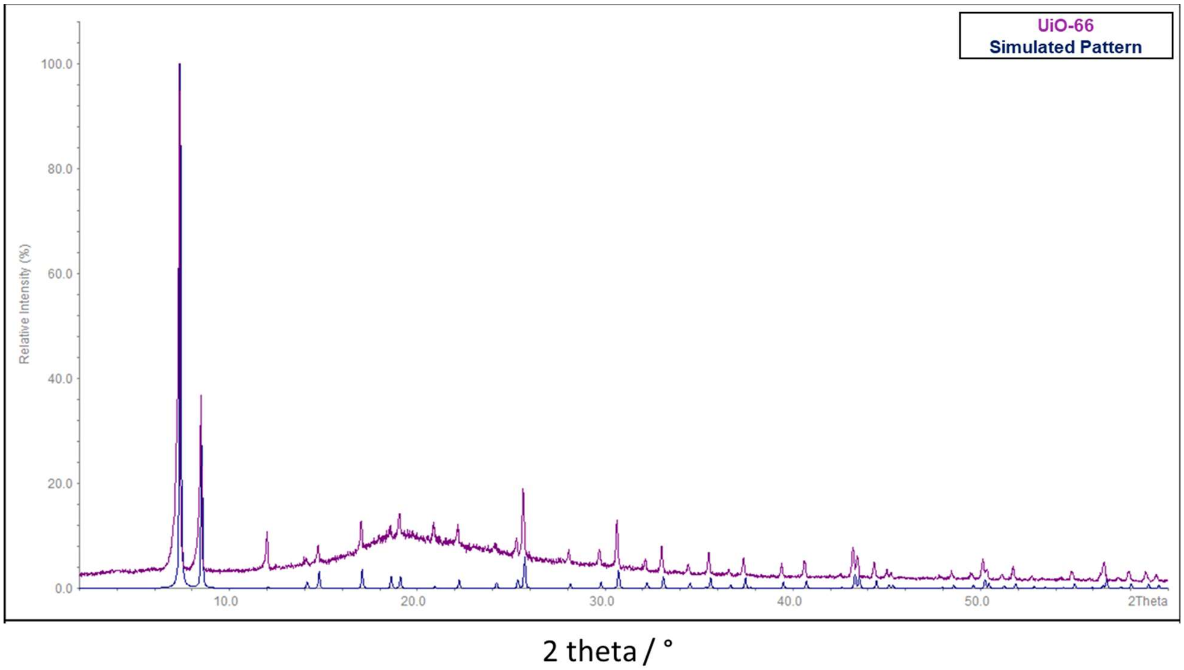

Figure S4. PXRD patterns of HKUST-1, MOF-303, CALF-20, [Cu-4py-Me], UiO-66 in comparison with simulated patterns based on single crystal data.

S6 FT-IR Spectra

Infrared spectra were recorded using a Bruker Vertex 80V FTIR spectrometer with KBr pellets, analysis was performed using OPUS 6.0. KBr pellets were prepared from 2 mg of the MOF sample and 200 mg of KBr. Pre- and post-treatment measurements were conducted after heating the samples to 100 °C for water molecule removal. Normalization and baseline correction were applied post-spectra acquisition. According to Figure S5, no pure ligand is present within the MOF pores.

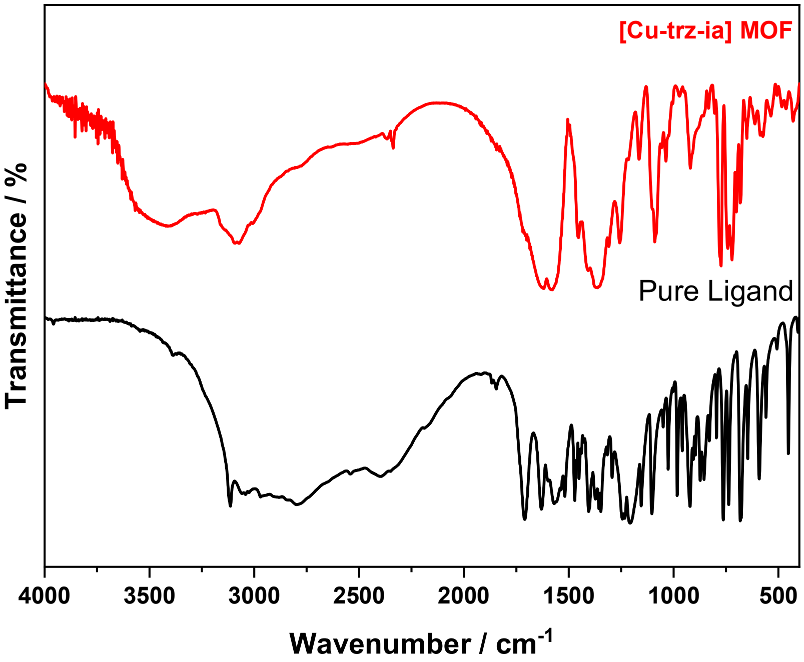

Figure S5. FT-IR spectra of [Cu_2(trz-ia)$_2$] and dimethylammonium-3-carboxy-5-(4H-1,2,4-triazol-4-yl) benzoate (pure ligand).

S7 Thermogravimetric Analysis

The TG-MS analyses were conducted employing corundum crucibles on a thermobalance (STA 449 F1 Jupiter, Netzsch) coupled with an Aeolos QMS 403C mass spectrometer[11]. The sample underwent heating at a rate of 10 K min^{-1}, reaching 600 °C, within a continuous flow of Argon (99.999%). The sample was solvent exchanged with methanol prior to the TG-DTA measurement.

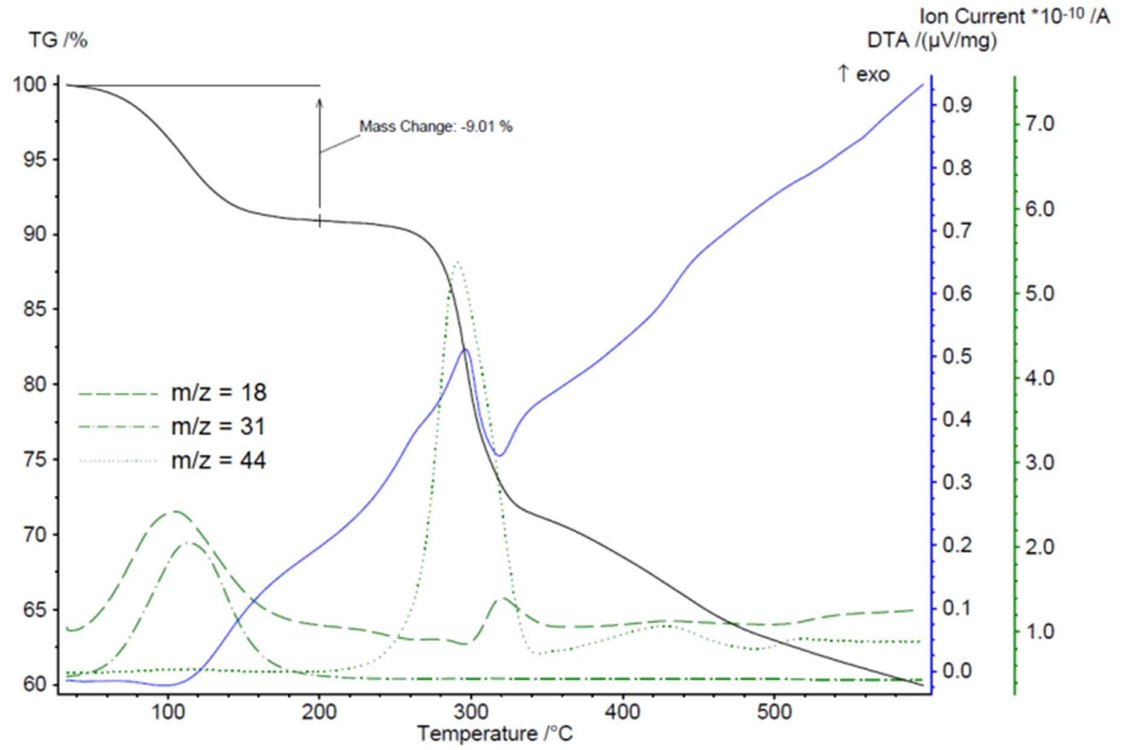

Figure S6. The TG-DTA-MS analysis showcases the evaporation of guest molecules (below 160 °C) and the decomposition of the framework (above 260 °C), as evidenced by the detected MS signals for $(H_2O)^+$ (m/z=18), $(MeO)^+$ (m/z=31), and $(CO_2)^+$ (m/z=44).

S8 X-ray Photoelectron Spectroscopy

In order to distinguish between Cu atoms with different oxidation states, Cu^{I} and Cu^{II}, in $[Cu_2(trz\text{-}ia)_2]$, XPS analysis was carried out on a photoelectron spectrometer from Thermo Fisher Scientific Instruments, UK, with monochromatic Al-K_α radiation as the X-ray source operating at 12 kV with 6 mA beam current. All spectra were calibrated with respect to adventitious carbon observed at 285 eV. A survey scan was performed on each sample before the elemental scans in order to identify every element that was present. Using the CASA XPS tool[12], the XPS spectra were deconvoluted using a Gaussian Lorentzian mix function with smooth linear background subtraction.

In all the XPS survey scans for $[Cu_2(trz\text{-}ia)_2]$, the spectra of each sample display the distinctive peaks from copper, CuLMM Auger, oxygen, nitrogen, and carbon signals as expected from the 1,2,4-triazolyl isophthalate linker. The accompanying CuLMM Auger peaks at 916.5 eV, typical for Cu^{I}, and at 917.2 eV, typical for Cu^{II} species, provide additional evidence that two different types of Cu are present.[13]

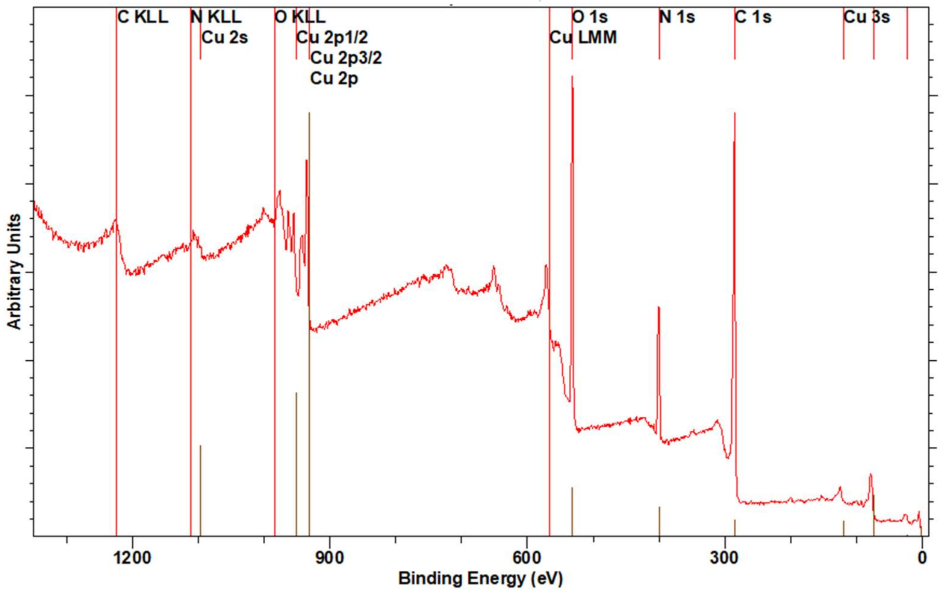

Figure S7. XPS survey scan spectra of $[Cu_2(trz\text{-}ia)_2]$

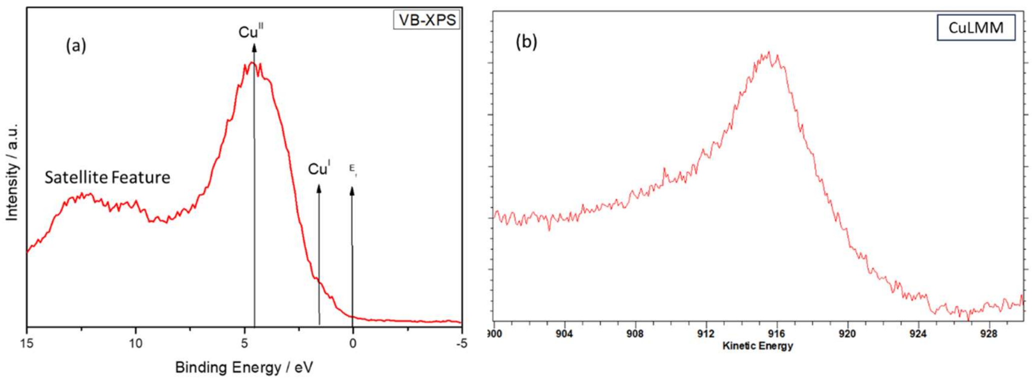

Figure S8. (a) X-ray Valence Band Cu spectra (b) Cu LMM Auger spectra of [Cu_2(trz-ia)$_2$]

S9. Electron Paramagnetic Resonance

Sample preparation: About 25 mg of solvent exchanged [Cu_2(trz-ia)$_2$] in methanol were placed in a quartz tube with an inner diameter of 3.8 mm. EPR studies for all [Cu_2(trz-ia)$_2$] samples were conducted in their solvated state, meaning they suspended in methanol.

cw EPR experiments: the X-band *cw* EPR spectra were recorded at temperatures ranging from 10 to 160 K using a Bruker EMXmicro spectrometer equipped with a Bruker ER4119HS cylindrical cavity and an ESR900 helium cryostat (Oxford Instruments). EPR spectral analysis was carried out using Easyspin[14] simulation package embedded in MATLAB. The spectral simulation for an anisotropic and isolated $S = 1$ system can be described as follows

$$\hat{H} = \beta_e \vec{B}\vec{\vec{g}}\hat{\vec{S}} + D\left[\hat{S}_z^2 - \frac{1}{3}S(S+1) + E\left(\hat{S}_x^2 + \hat{S}_y^2\right)\right] \quad (1)$$

where β_e denotes Bohr's magneton and $\vec{\vec{g}}$ describes the Cu^{II}-Cu^{II} pair *g*-tensor. $\vec{B}$ is the external magnetic field. Both *D* (axial) and *E* (rhombic) represent zero-field splitting (zfs) parameters. Here the zfs tensor and the $\vec{\vec{g}}$ tensor are assumed to be coaxial and $\hat{\vec{S}}$ is the electron spin operator with S =1.

On the other hand, the uncoupled Cu^{II} system can be simulated as follows

$$\hat{H} = \beta_e \vec{B}\vec{g}\hat{\vec{S}} + \hat{\vec{S}}\vec{A}^{\mathrm{Cu}}\hat{\vec{I}}^{\mathrm{Cu}} \quad (2)$$

The first term describes the electron Zeeman interaction of the electron spin S = ½ with its spin vector operator $\hat{\vec{S}}$ and *g*-tensor of the Cu^{II} ion ($\vec{g}$) and the external magnetic field $\vec{B}$. The second term implements the hyperfine (hf) interaction between the interaction tensor $\vec{A}^{\mathrm{Cu}}$ of the

electron spin with the ^{63,65}Cu nuclear spin $I_{Cu} = 3/2$ where $\hat{\vec{I}}^{Cu}$ is the corresponding nuclear spin vector operator.

S9.1 Calculation of mononuclear defect species in [Cu_2(trz-ia)$_2$]

The *cw* EPR temperature-dependent dataset, specifically the signal amplitude of the $B_{x2,y2}$ line transition is used as a function of temperature to estimate the isotropic exchange coupling constant (J) of the antiferromagnetically coupled Cu^{II}-Cu^{II} pairs according to the Bleaney-Bowers equation[15]:

$$\chi = \mu_0 \frac{2\beta_e^2 g^2}{k_B T} (3 + e^{-J/K_B T})^{-1} \tag{3}$$

where $\mu_0 = 4\pi \cdot 10^{-7}$ T·m·A^{-1} is the permeability of vacuum, g is an average of the principal values of the $\boldsymbol{g}$-tensor and β_e is the Bohr magneton. In addition, we may consider that the magnetic susceptibility (χ) of the dinuclear Cu^{II}_2 paddle wheel species is proportional to the relative intensity of the $S = 1$ spectrum.

The ratio of the EPR signal intensities between uncoupled Cu^{II} and dinuclear Cu^{II}_2 paddle wheel species (I_M/I_{PW}) can be expressed as[11]

$$\frac{I_M}{I_{PW}} = \frac{\chi_M}{\chi_{PW}} = \frac{1}{8}\frac{N_M}{N_{PW}}\left[3 + \exp\left(\frac{-J}{k_B T}\right)\right] \tag{4}$$

By taking J of -234 cm^{-1} obtained from the fitting of temperature-dependent *cw* EPR data (**Figure S9 (b) , (c**), the intensity ratio of the signals of the uncoupled Cu^{II} species and Cu^{II}_2 dinuclear paddle wheel units (I_M/I_{PW}) can be estimated by fitting the weight (contribution) for each individual species (i.e., uncoupled Cu^{II} species (I_M) and Cu^{II}_2 dinuclear paddle wheel units

(I_{PW}) to the overall spectral simulation of the experimental spectra at 120 K, yielding the ratio of N_M/N_{PW} = 0.15 or 15 %.

Table S1. Simulation parameters for Cu^{II} related species in the $[Cu_2(trz\text{-}ia)_2]$ MOF.

Parameter	dinuclear Cu_2^{II} paddle-wheel species	uncoupled Cu^{II} species
Spin	$S = 1$	$S = 1/2$
g ($g_{xx,yy}$,g_{zz})	2.080, 2.355	2.080, 2.355
A ($A_{xx,yy}$,A_{zz})	50 MHz, 200 MHz	50 MHz, 400 MHz
D and E	0.370 cm^{-1} and 0 cm^{-1}	-
D strain	0.033 cm^{-1}	-

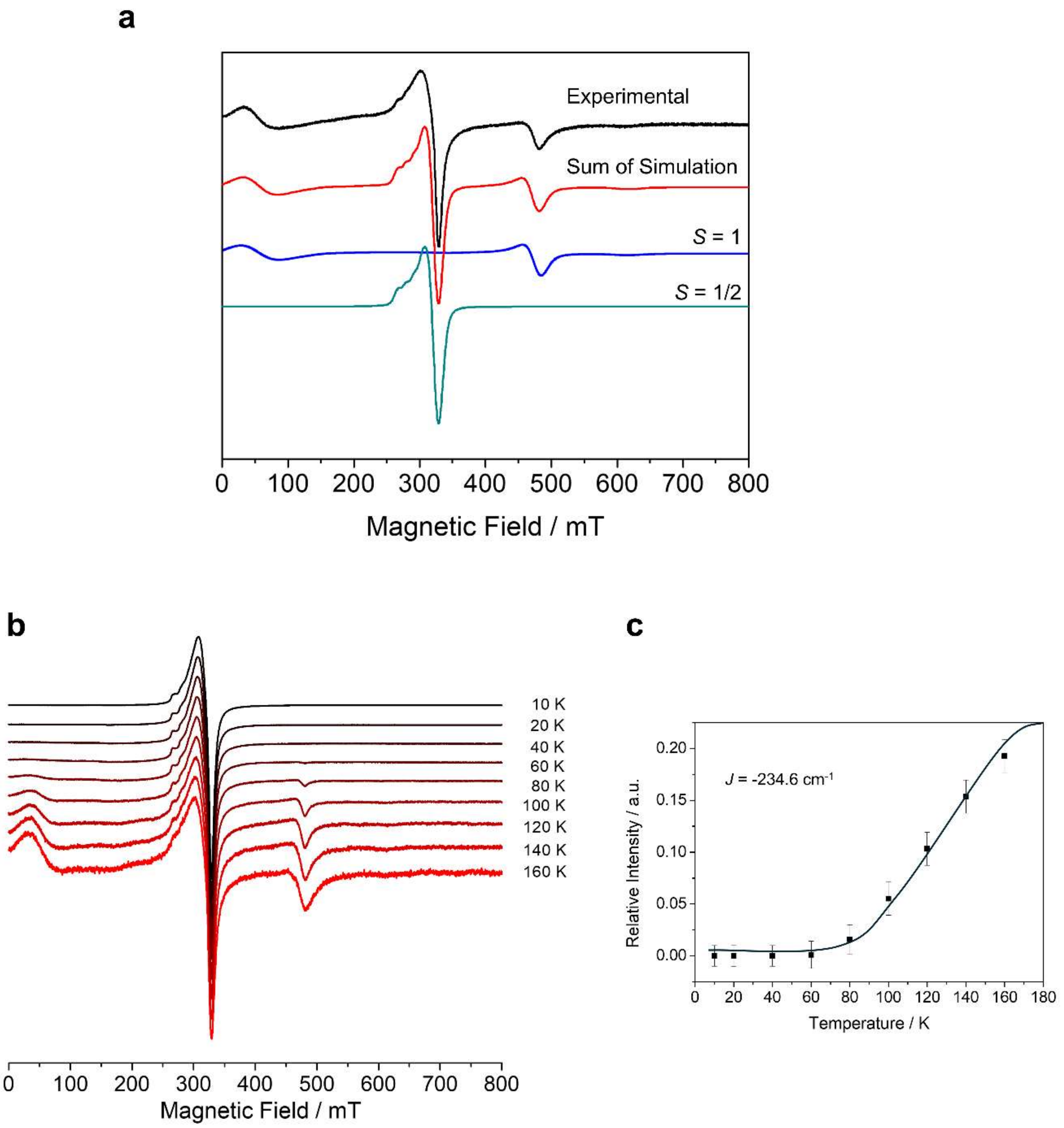

Figure S9. (a) Deconvolution of *cw* X-band EPR spectra of [Cu_2(trz-ia)$_2$] at 120 K according to the spectral simulation of S = 1 and S = 1/2 species. (b) temperature-dependent *cw* EPR spectra and (c) Temperature dependence of the normalized EPR intensity of the S = 1 species using the $B_{x2,y2}$ signal. Solid curve is the best fit to the Bleaney–Bowers equation.

S10 Gas Adsorption

High-resolution, low-pressure sorption isotherms ranging up to 1 bar were recorded at temperatures between 67 K and 87 K using a Belsorp-max (Microtrac MRB) equipped with a closed-cycle helium cryostat. Prior to the measurement, all samples (50-100 mg) underwent overnight activation in a dynamic vacuum. The measurement spanned a pressure range from 0 to 100 kPa, data analysis utilized BEL Master 6.3.0.0 software for Belsorp-max measurements. H_2 adsorption experiments for HKUST-1, MOF-303, CALF-20, UiO-66, [Cu-4py-Me] MOFs were performed on a Belsorp-max G (Microtrac, Version 1.1.0) at 77 K using liquid nitrogen and at 87 K using a *cryoTune* from 3P Instruments.

For all measurement we employed 99.998% pure helium gas for dead volume determination, while high-purity N_2, CO_2, H_2, D_2 gas (99.998%) was used for physisorption.

N_2 adsorption experiments at 77 K were performed on a Belsorp-max G (Microtrac, Version 1.1.0). CO_2 gas adsorption at 298 K and 195 K up to 100 kPa, was measured using the same instrument equipped with a thermostat and BELmaster version 7.3.2.0 software for further analysis. CO_2 sorption experiments at 195 K were performed using dry ice in ethanol as cooling baths.

S10.1 Pore Size Distribution

The pore size distribution analysis was conducted using a CO_2 adsorption isotherm measured at 298 K up to 100 kPa, utilizing the computer simulation method known as Grand Canonical Monte Carlo (GCMC).

The parameters for the analysis included an Interpolated Curve (Three-Dimensional Spline Curve), Kernel Method: GCMC, Pore Model: Cylinder, and PSD Fitting parameter used:

Tikhonov Regulation. These settings allowed for a comprehensive assessment of the pore size distribution using advanced computational methods.

S10.2 Calculation of the number of H_2 molecules adsorbed per paddle wheel unit of $[Cu_2(trz\text{-}ia)_2]$ and per unit cell.

- The unit cell of $[Cu_2(trz\text{-}ia)_2]$ contains two formula units (Z = 2) of $[Cu_2(trz\text{-}ia)_2]$ with a formula weight of $M_m = 589.42\ g\ mol^{-1}$.
- The H_2 gas uptake for [Cu-trz-ia] at 67 K and 100 kPa amounts to 0.0123 mol g^{-1}.
- Thus, the number of H_2 molecules adsorbed per paddle wheel unit is $0.0123\ \frac{mol_{H2}}{g} \cdot 589.42\ \frac{g}{mol} = 7.25$, accordingly, one unit cell of $[Cu_2(trz\text{-}ia)_2]$ adsorbs $7.25 \cdot Z = 14.5$ molecules of H_2.

Temperature (K)	Uptake (mol g^{-1})	H_2 molecules per paddle-wheel	H_2 molecules per unit cell
67	0.0123	7.25	14.5
77	0.0105	6.15	12.3
87	0.0854	5.0	10.0

S10.3 Calculation of volumetric hydrogen uptake and hydrogen density

The gravimetric H_2 uptake at 77 K and 100 kPa is 0.0105 mol $g^{-1} \cdot 2.0\ g\ mol^{-1} = 21.1\ mg\ g^{-1}$. With the single crystal density (obtained from CIF file) of $\rho_{calc} = 1.086\ g\ cm^{-3}$ the pore volume of 0.48 mL g^{-1} is calculated using Gurvich Equation with high pressure CO_2 adsorption data measured at 298 K and 10 MPa.[3]

Volumetric uptake: 21.1 mg $g^{-1} \cdot$ 1.086 g cm^3 = 23 mg cm^{-3} or 23 mg mL^{-1}

The gas density inside the pore can be calculated by gravimetric H_2 uptake divided by the pore volume: $\frac{21.1\ mg/g}{0.48\ mL/g}$ = 43.9 mg mL^{-1}

S10.4 Comparison of hydrogen adsorption data for various MOFs obtained from NIST Database.

HKUST-1[4], UiO-66[5], MOF-303[6], [Cu-4-py-Me][7] and CALF-20[8] were synthesized according to procedures published in literature (Section S3). The results of hydrogen adsorption measurements are reported below including their respective references. Remaining data were taken from the NIST database with their respective references cited accordingly.

Table S2. Data for several MOF adsorption isotherms, acquired at 77 K and 100 kPa pressure.

MOF	Q_{ads} for H_2 (low coverage)	Pore Size	H_2 adsorbed at 5 kPa (mmol g^{-1})	H_2 adsorbed at 100 kPa (mmol g^{-1})	Reference
MOF-5	3.8 kJ / mol	1.1 nm to 1.5 nm	1.56	6.9	[16]
FJI-1	4.6 kJ / mol	1.4 nm	0.51	4.9	[17]
MOF-1c	8.8kJ / mol	0.47 to 0.57 nm	2.73	7.2	[18]
Cu-Bdc-OH	8.4 kJ / mol	0.3 nm	1.76	6	[19]
Mg-Formate	7.0 kJ / mol	0.34 nm	0.48	4.9	[20]
UTSA-33	-	0.48 nm to 6.5 nm	1.76	7.2	[21]

UTSA-36a	-	0.31 to 4.4 nm	2	5.4	[22]
[Cu-4py-Me]	6.5 kJ / mol	0.55 nm	4.7	15.4	[7]
MOF-508a	-	0.4 nm	1.71	3.9	[23]
Co-Formate	8.3 kJ / mol	0.5 to 0.6 nm	1.78	3.7	[24]
CDC	8.5 kJ / mol	0.67 nm	2.2	10.6	[25]
CALF-20	8.4 kJ / mol	0.34 nm to 4.4 nm	2.44	5.7	This work
HKUST-1	6.5 kJ / mol	1.4 nm to 0.5 nm	3.2	10.4	This work
UiO-66	8.1 kJ / mol	0.43 to 0.51 nm	2.19	8.2	This work
MOF-303	7.0 kJ / mol	0.6 nm	2.3	10.1	This work
$[Cu_2(trz\text{-}ia)_2]$	9.7 kJ / mol	0.34 to 0.53 nm	4.7	10.5	This work

S10.5 Isosteric heat of adsorption

The calculation of the heat of adsorption, Q_{ads}, was executed using the Clausius-Clapeyron approach, for more details please refer to the previously published work.[26]

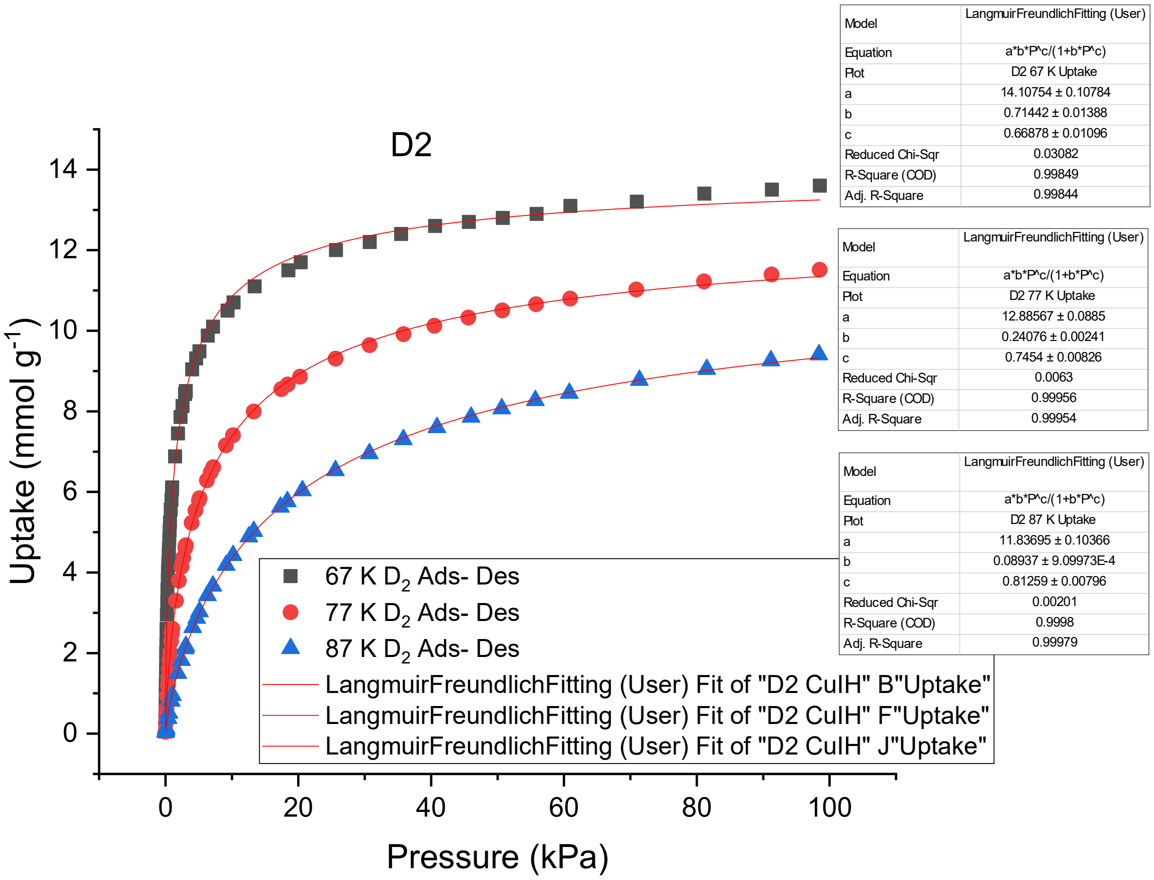

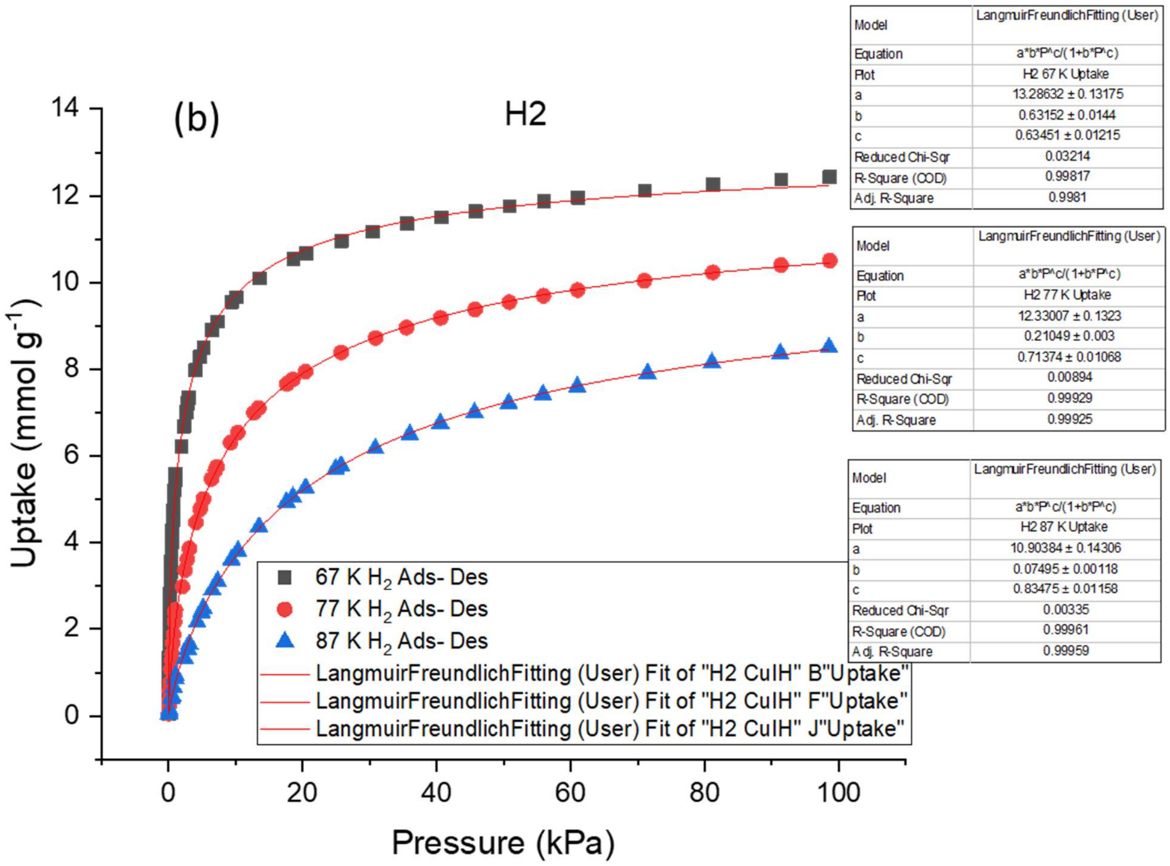

Figure S10. Langmuir-Freundlich fit for D_2 and H_2 isotherms of [Cu_2(trz-ia)$_2$] at 67, 77 and 87 K.

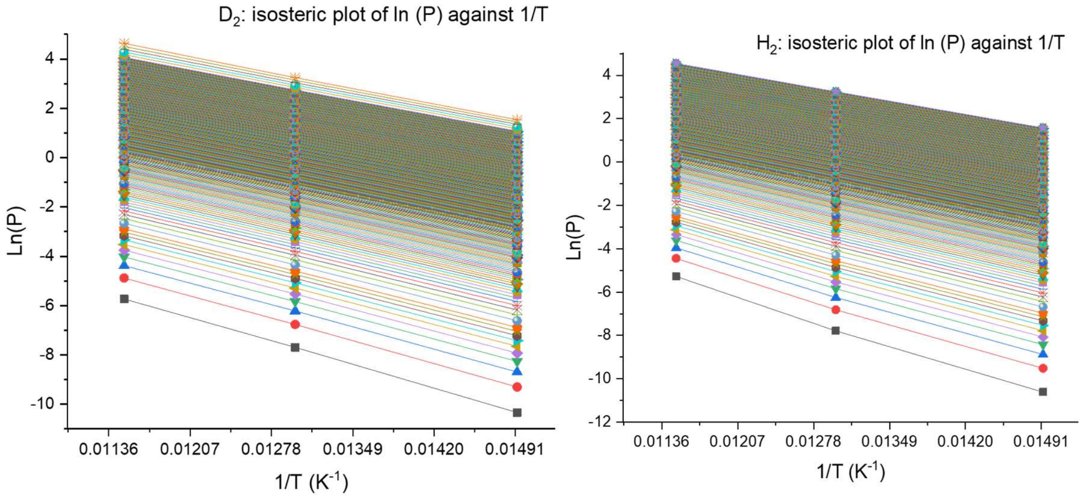

Figure S11. Isosteric plot of ln (p) against 1/T for different loadings n (in mmol g^{-1}) of [Cu_2(trz-ia)$_2$] with D_2 and H_2 gas at 67, 77 and 87 K.

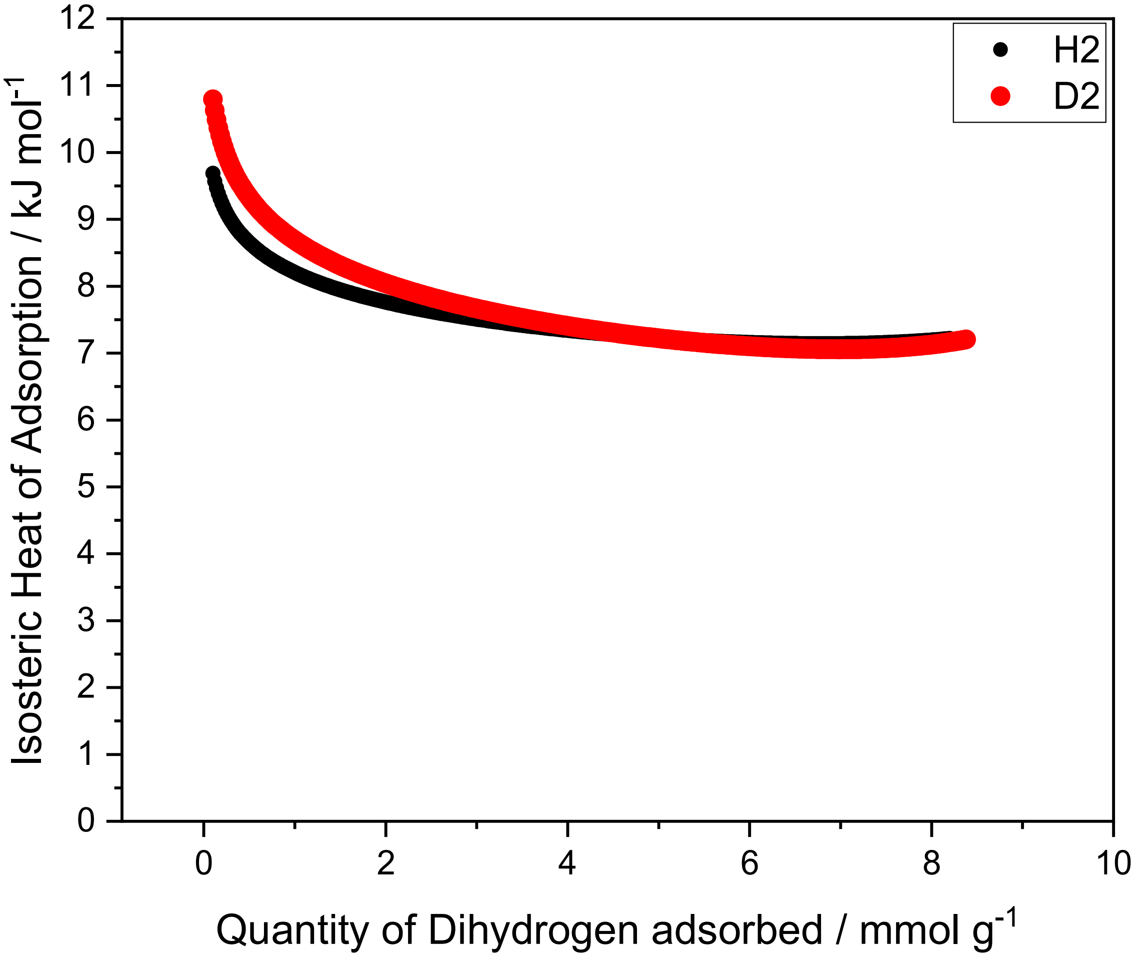

Figure S12. Langmuir-Freundlich fits and Clausius-Clapeyron equation were used to calculate Q_{ads} for D_2 (red) and H_2 (black) adsorption isotherms on [Cu_2(trz-ia)$_2$] samples at 67, 77 and 87 K, respectively.

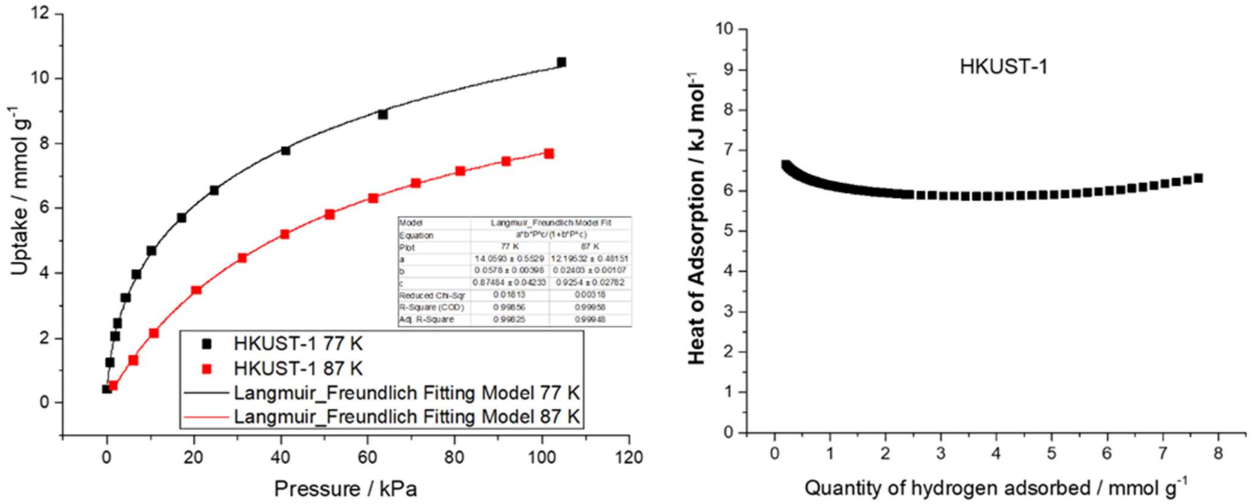

Figure S13. H_2 gas adsorption isotherm, Langmuir-Freundlich fit, and heat of adsorption for HKUST-1. Symbols – experimental data, lines – fit based on Langmuir-Freundlich model.

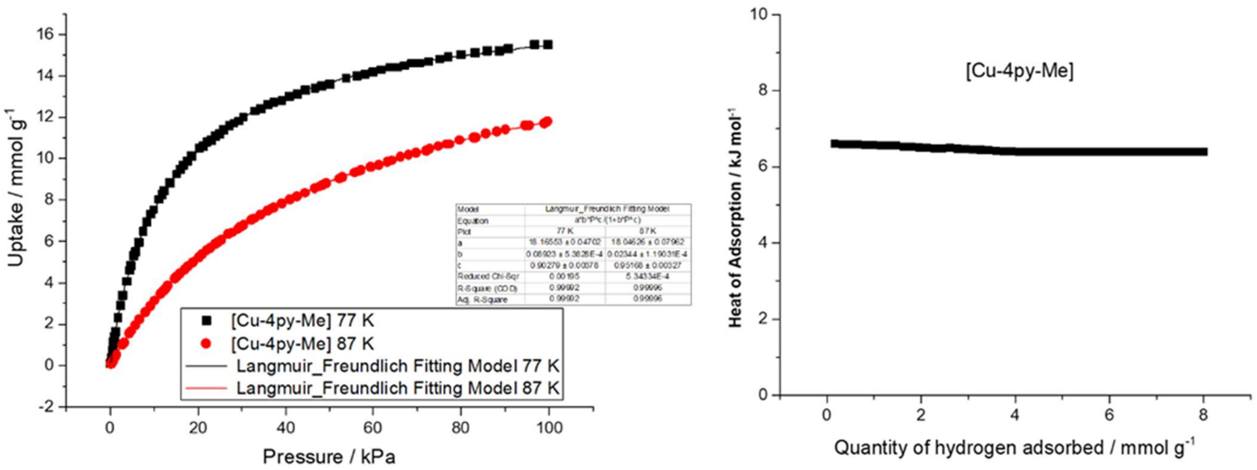

Figure S14. H_2 gas adsorption isotherm, Langmuir-Freundlich fit, and heat of adsorption for [Cu-4py-Me]. Symbols – experimental data, lines – fit based on Langmuir-Freundlich model.

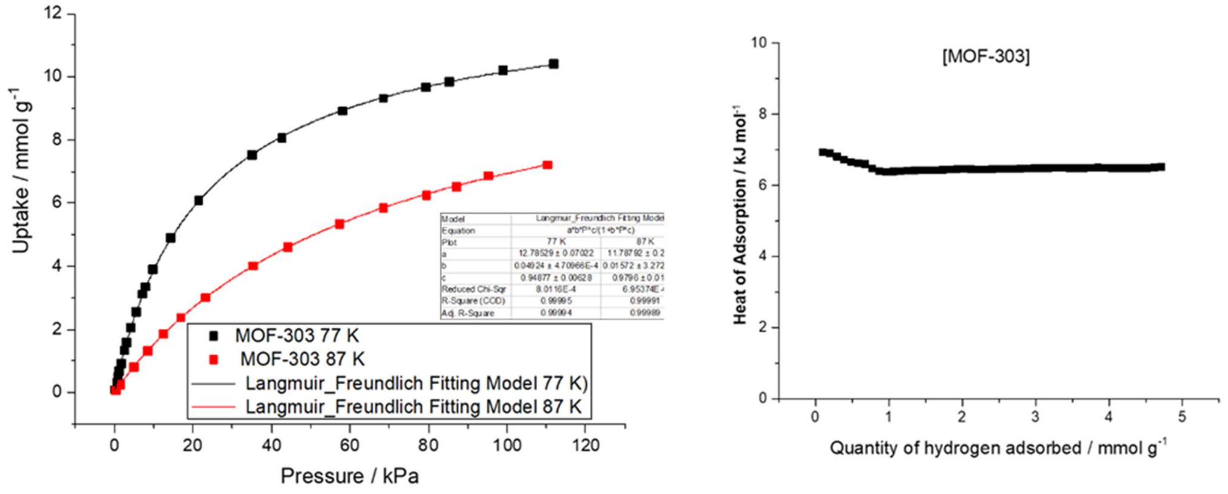

Figure S15. H_2 gas adsorption isotherm, Langmuir-Freundlich fit, and heat of adsorption for MOF-303. Symbols – experimental data, lines – fit based on Langmuir-Freundlich model.

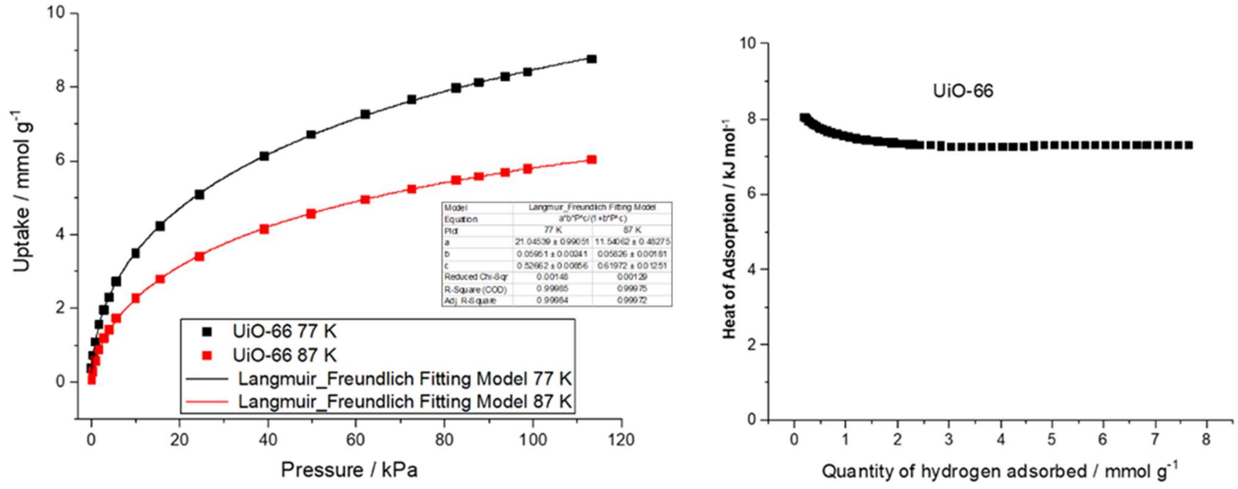

Figure S16. H_2 gas adsorption isotherm, Langmuir-Freundlich fit, and heat of adsorption for UiO-66. Symbols – experimental data, lines – fit based on Langmuir-Freundlich model.

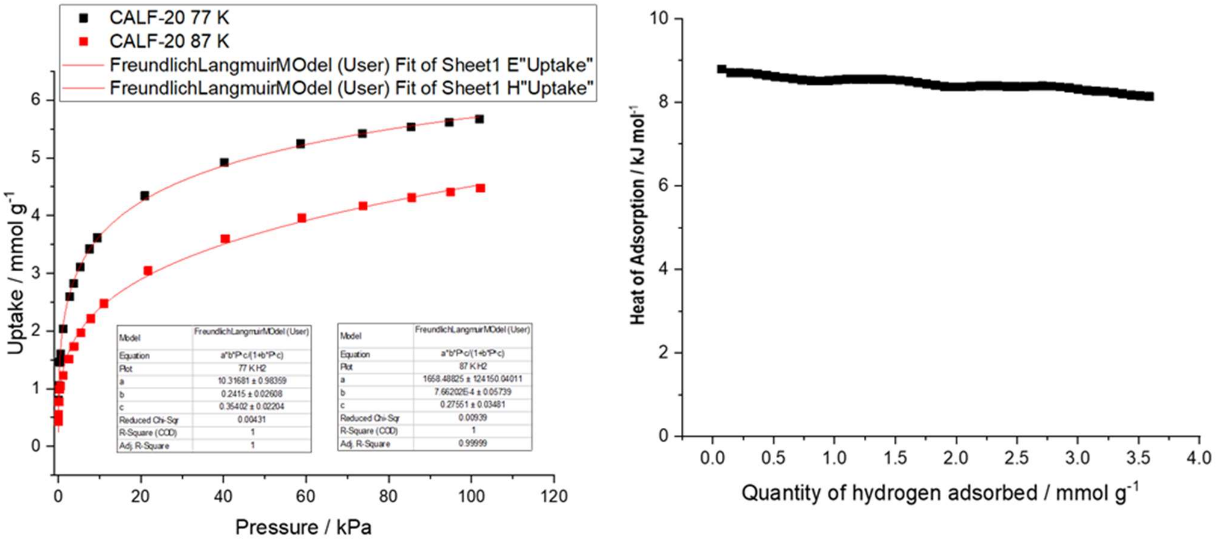

Figure S17. H_2 gas adsorption isotherm, Langmuir-Freundlich fit, and heat of adsorption for CALF-20. Symbols – experimental data, lines – fit based on Langmuir-Freundlich model.

S10.6. N_2 and CO_2 Sorption studies

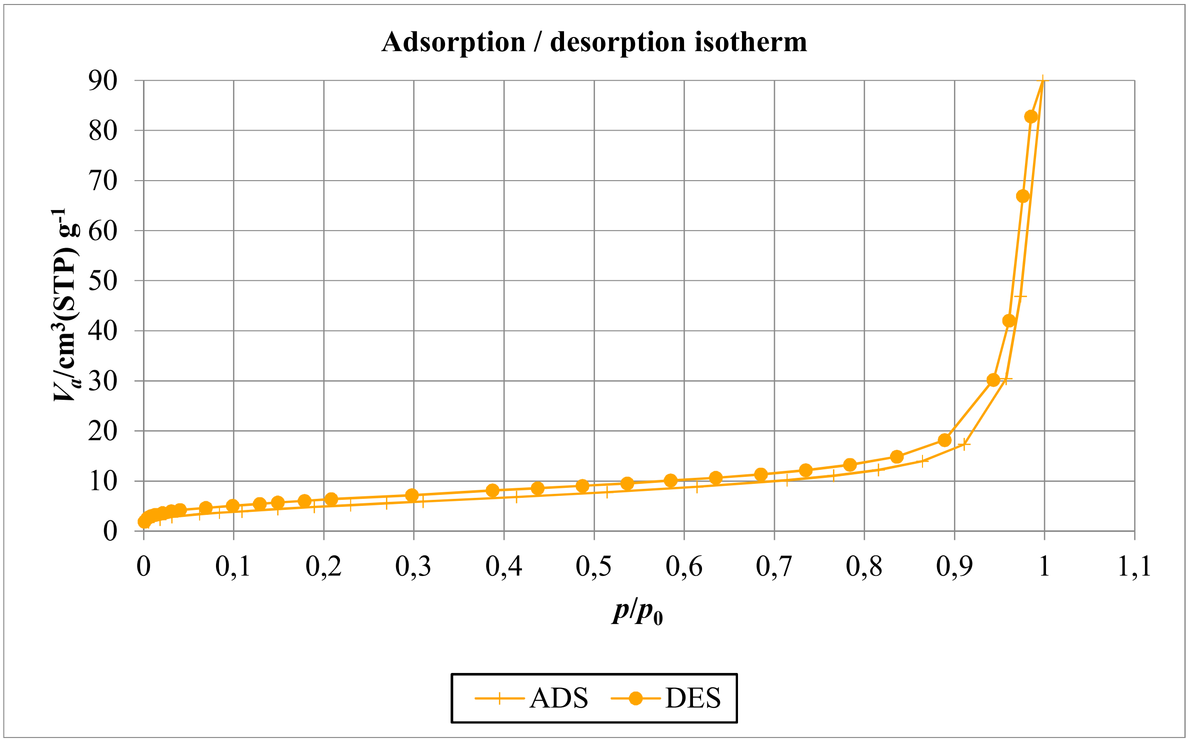

Figure S18. N_2 adsorption-desorption isotherms of [Cu_2(trz-ia)$_2$] recorded at 77 K up to 100 kPa.

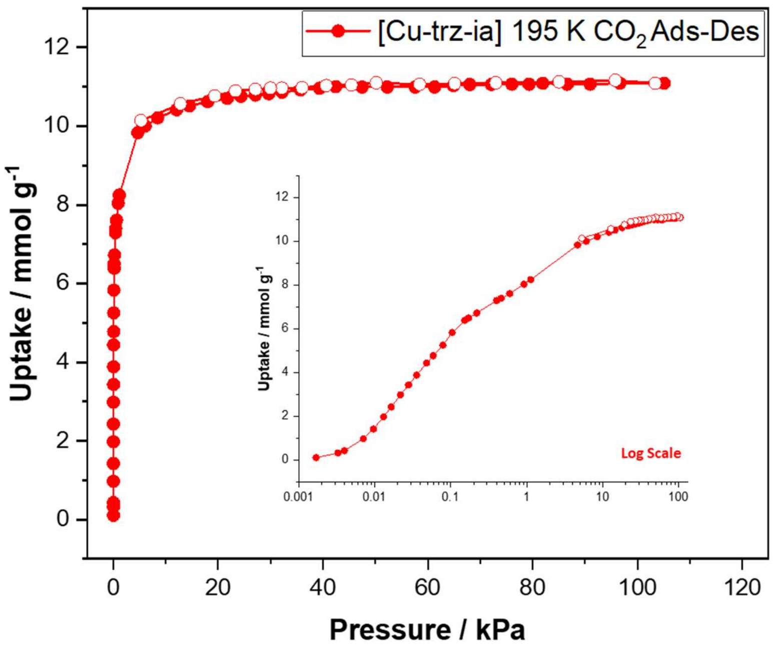

Figure S19. CO_2 adsorption-desorption isotherms of [Cu_2(trz-ia)$_2$] recorded at 195 K up to 100 kPa.

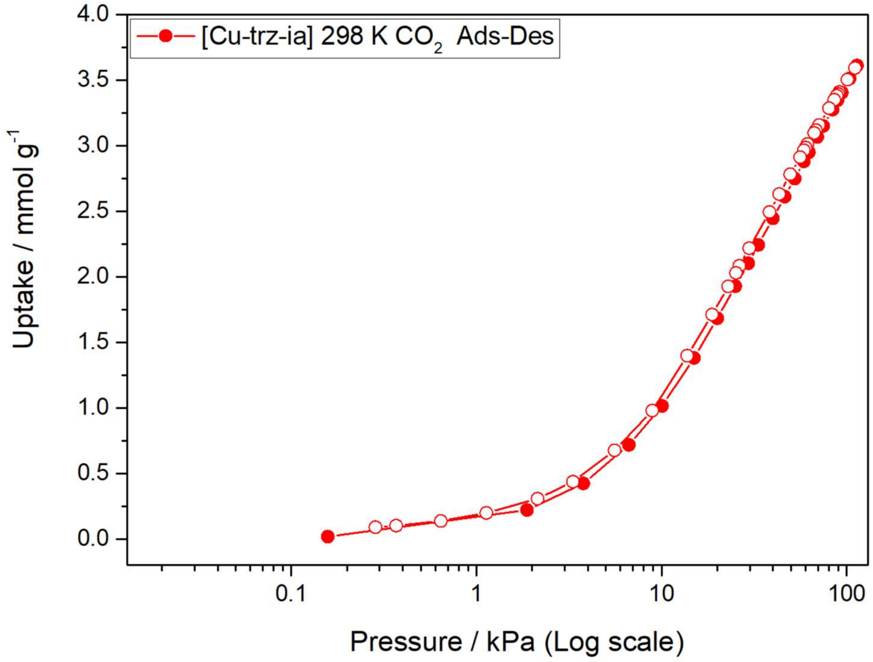

Figure S20. CO_2 adsorption-desorption isotherm of [Cu_2(trz-ia)$_2$] recorded at 298 K up to 100 kPa.

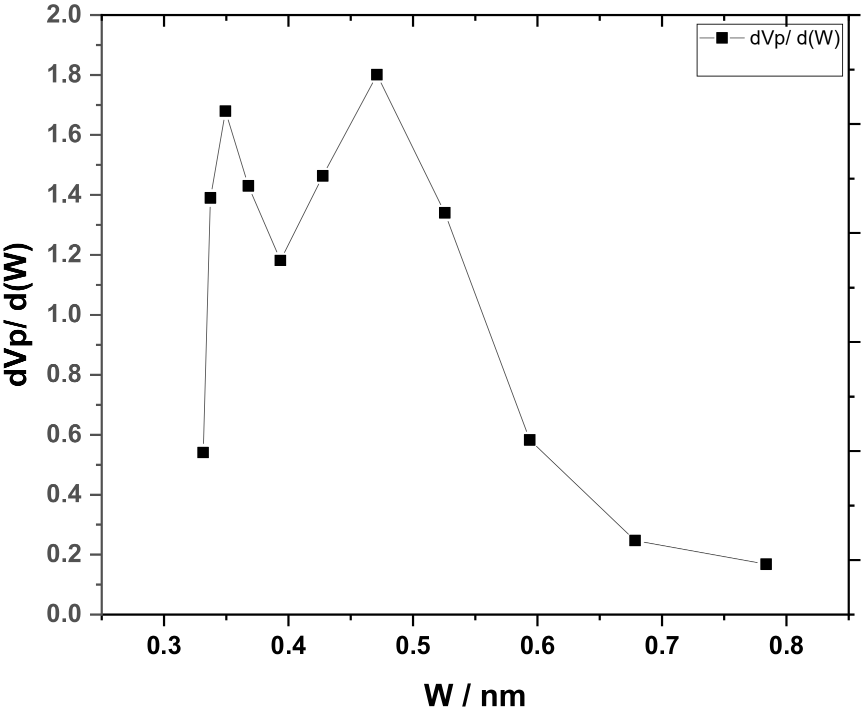

Figure S21. PSD of $[Cu_2(trz\text{-}ia)_2]$ calculated using GCMC method from CO_2 isotherm recorded at 298 K up to 100 kPa.

S11 Thermal Desorption Spectroscopy (TDS)

A Cryogenic Thermal Desorption Spectroscopy system was employed to investigate the sample's distinct preferential adsorption sites and evaluate its isotope separation characteristics. About 3-4 mg of sample was taken inside the device and it was activated overnight under ultra-high vacuum (10^{-5} mbar) to remove all the adsorbed species.

TDS procedure details for pure H_2 and D_2: The sample is subjected to a 1.0 kPa environment of D_2 or H_2. The sample is then rapidly cooled to the boiling point of the adsorbed gas, and any gas molecules that were not adsorbed are removed by vacuum. Finally, a linear heating ramp (0.1 $K \cdot s^{-1}$) is used. A quadrupole mass spectrometer is used to continuously detect desorbing gas, recognizing a pressure increase in the sample chamber as gas desorbs. The area under the desorption peak is proportional to the amount of gas desorbing, which may be calculated when the TDS equipment has been carefully calibrated using $Pd_{95}Ce_5$ alloy.[27]

TDS specifications for hydrogen isotope separation: At room temperature, the sample chamber is in ultrahigh vacuum (UHV) and cools to the desired temperature of 25 K, 30 K or 40 K, respectively. The sample is then subjected to a prescribed 1:1 D_2:H_2 equimolar mixture (10.0 kPa) for ten minutes. After loading a 1:1 D_2:H_2 mixture at the specified exposure temperature and pressure, the gas molecules that had not been adsorbed were removed by vacuum. The sample is then rapidly cooled to the boiling point of the adsorbed gas. Finally, a linear heating ramp (0.1 $K \cdot s^{-1}$) activates thermal desorption. A quadrupole mass spectrometer constantly detects the desorbing gas, recognizing a pressure increase in the sample chamber as the gas desorbs. The area under the desorption peak is related to the amount of gas being desorbed.

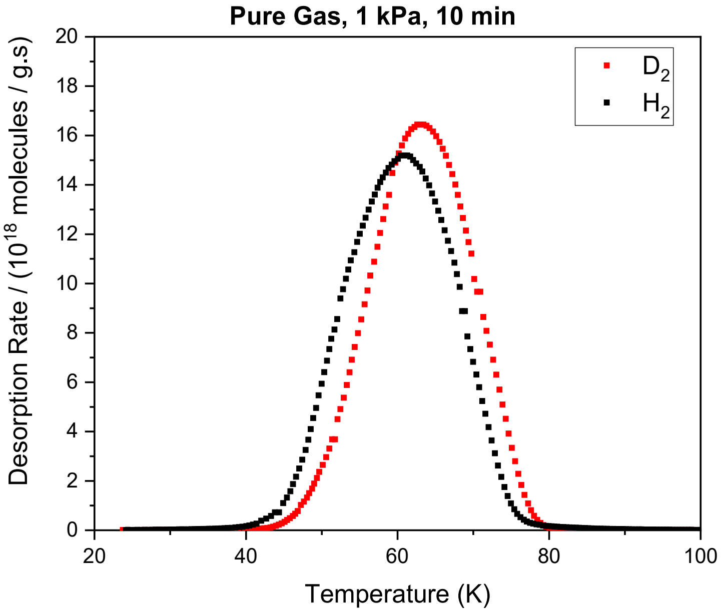

Figure S22. D_2 (red) and H_2 (black) TDS curves for [Cu_2(trz-ia)$_2$] after room temperature exposure to 1.0 kPa of pure H_2 and D_2 gas and then cooling to 20 K.

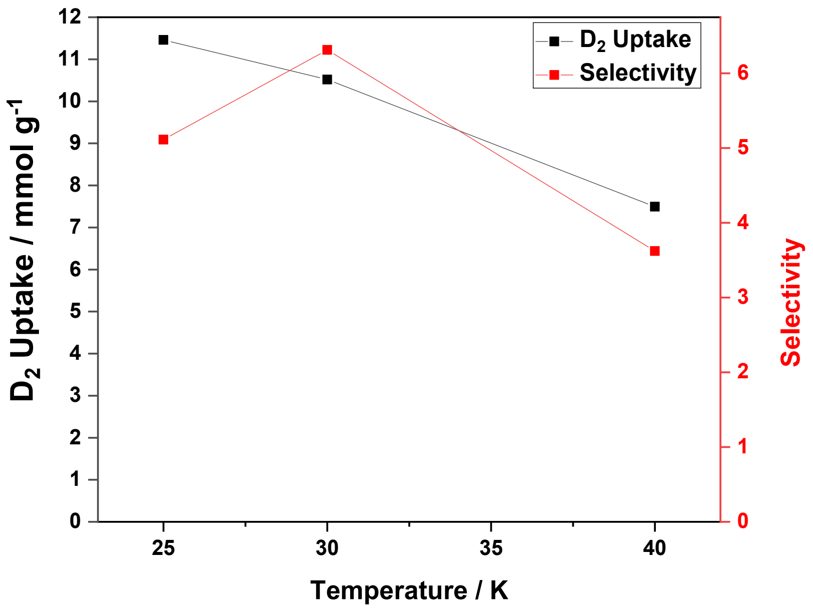

Figure S23. Comparison of D_2 uptake (black, left axis) and selectivity (red, right axis) for D_2 over H_2 as a function of temperature in the studied [Cu_2(trz-ia)$_2$].

S12 Scanning Electron Microscopy (SEM)

The scanning electron microscope images of a [Cu_2(trz-ia)$_2$] sample were acquired on a Leo Gemini from Zeiss, Modell 7426 using Oxford instruments at 20 kV. SEM measurements were conducted on a powder sample to examine the morphology after solvent evaporation. The particle size falls within the range of 1 µm to 2 µm. The crystallites exhibit a non-uniform surface and agglomeration of particles.

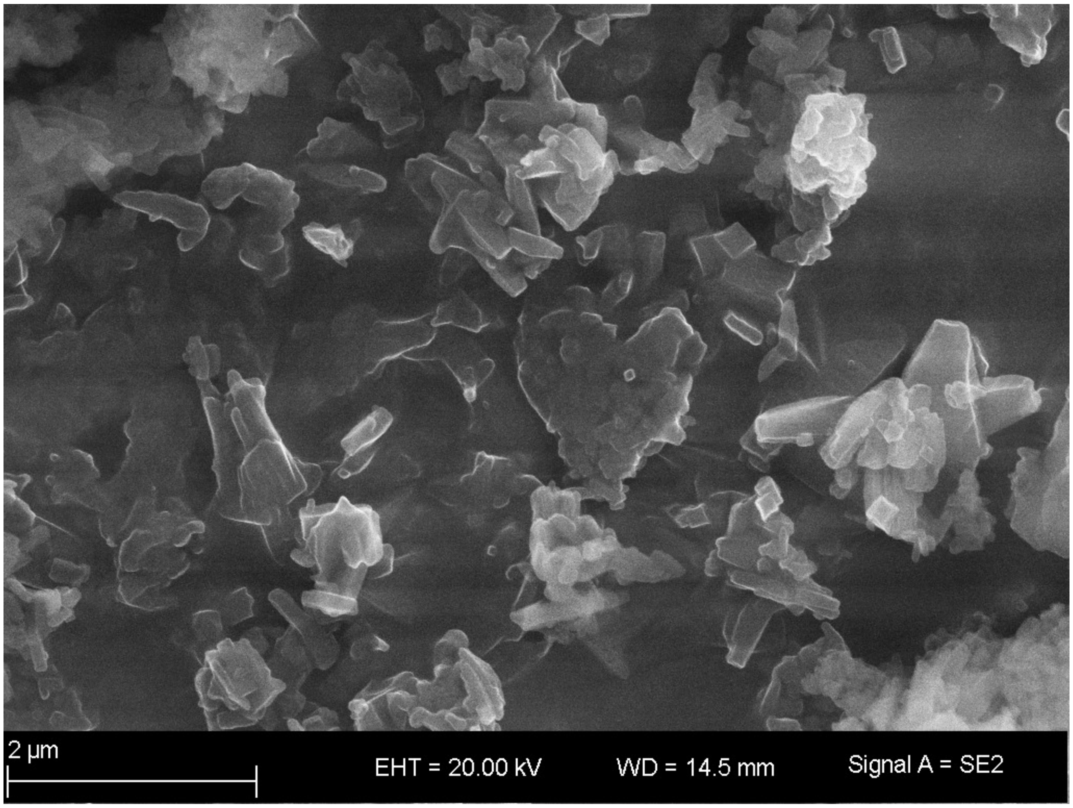

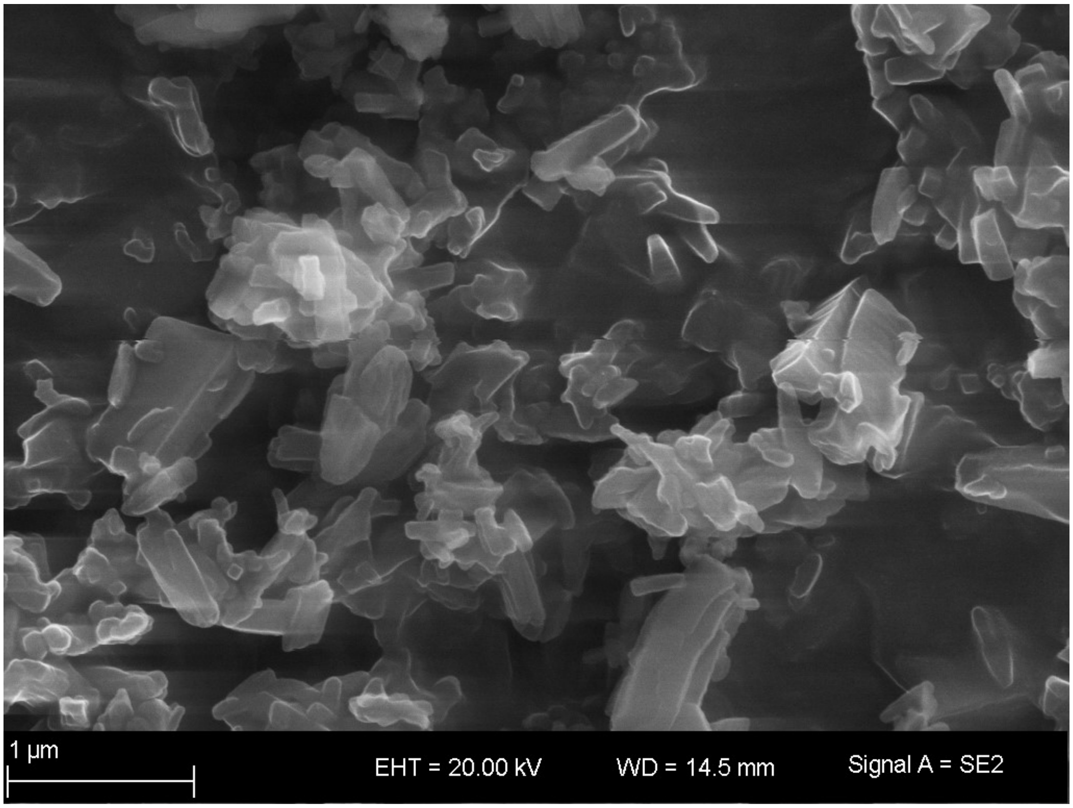

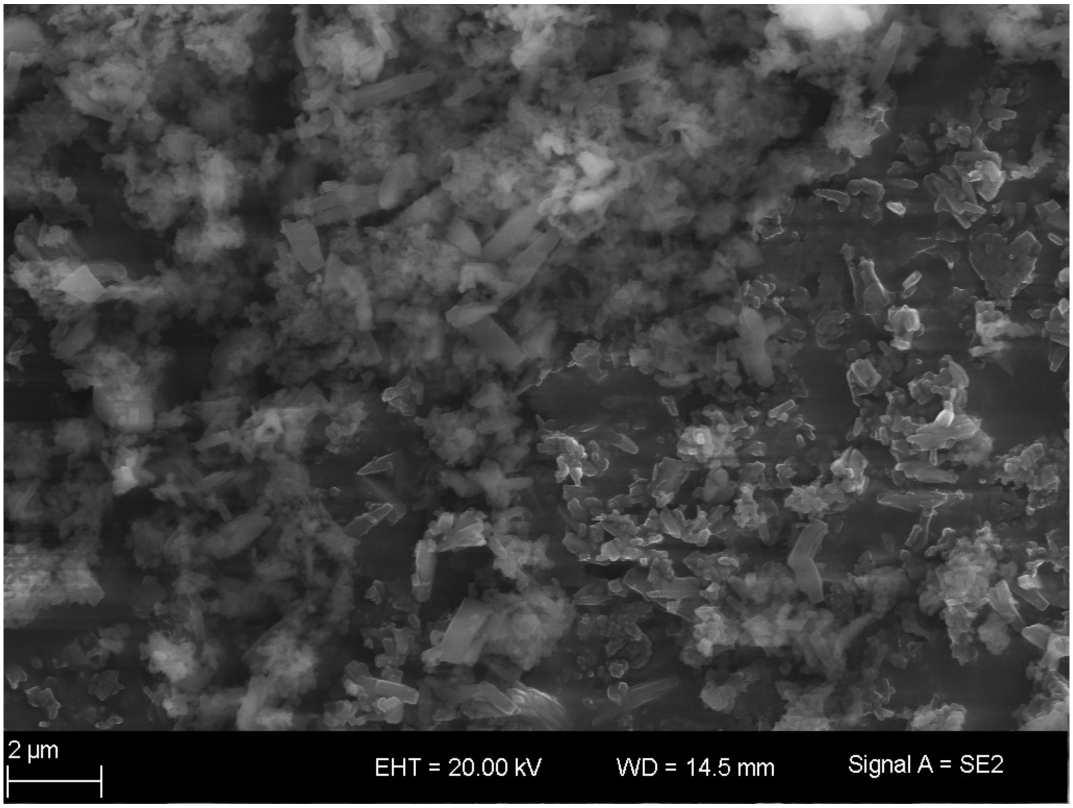

Figure S24. SEM images of $[Cu_2(trz\text{-}ia)_2]$

S13 Elemental analysis

All samples were solvent exchanged with MeOH and evacuated before the analysis. CHN elemental analyses were carried out on a VARIO EL analyzer (Elementar). The Cu content was determined by ICP-OES analysis performed on an Optima 8000 instrument (Perkin Elmer). For this, the sample was dissolved in nitric acid.

Table S3. Elemental analysis of [Cu_2(trz-ia)$_2$]

Name	Formula	Cu (ICP-OES)	C	N	H
[Cu_2(trz-ia)$_2$]	calculated $C_{20}H_{10}N_6O_8Cu_2$	21.56	40.75	14.26	1.71
[Cu_2(trz-ia)$_2$]·H_2O	calculated $C_{20}H_{12}N_6O_9Cu_2$	20.91	39.55	13.84	1.99
	measured	20.3	39.9	13.0	2.03

S14 References

1. Bartlett, R. K.; Humphrey, I. R. Transaminations of N,N-Dimethylformamide Azine. *J. Chem.Soc.C:Org.***1967**,*1664*. https://doi.org/10.1039/j39670001664.
2. Lässig, D.; Lincke, J.; Krautscheid, H. Highly Functionalised 3,4,5-Trisubstituted 1,2,4-Triazoles for Future Use as Ligands in Coordination Polymers. *Tetrahedron Lett.* **2010**, *51* (4), 653–656. https://doi.org/10.1016/j.tetlet.2009.11.098.
3. Kobalz, M.; Lincke, J.; Kobalz, K.; Erhart, O.; Bergmann, J.; Lässig, D.; Lange, M.; Möllmer, J.; Gläser, R.; Staudt, R.; Krautscheid, H. Paddle Wheel Based Triazolyl Isophthalate MOFs: Impact of Linker Modification on Crystal Structure and Gas Sorption Properties. *Inorg. Chem.* **2016**, *55* (6), 3030–3039. https://doi.org/10.1021/acs.inorgchem.5b02921.
4. Madden, D. G.; O'Nolan, D.; Rampal, N.; Babu, R.; Çamur, C.; Al Shakhs, A. N.; Zhang, S.-Y.; Rance, G. A.; Perez, J.; Maria Casati, N. P.; Cuadrado-Collados, C.; O'Sullivan, D.; Rice, N. P.; Gennett, T.; Parilla, P.; Shulda, S.; Hurst, K. E.; Stavila, V.; Allendorf, M. D.; Silvestre-Albero, J.; Forse, A. C.; Champness, N. R.; Chapman, K. W.; Fairen-Jimenez, D. Densified HKUST-1 Monoliths as a Route to High Volumetric and Gravimetric Hydrogen Storage Capacity. *J. Am. Chem. Soc.* **2022**, *144* (30), 13729–13739. https://doi.org/10.1021/jacs.2c04608.
5. Duncan, J.; Sengupta, D.; Bose, S.; Kirlikovali, K. O.; Farha, O. K. Defect-Induced Confinement in Zirconium Metal-Organic Frameworks for Enhanced Hydrogen Adsorption. *Sustainable Chemistry for the Environment* **2023**, *3*, 100032. https://doi.org/10.1016/j.scenv.2023.100032.
6. Kim, H.; Jee, S.; Park, J.; Jung, M.; Muhammad, R.; Choi, K.; Oh, H. High D_2/H_2 Selectivity Performance in MOF-303 under Ambient Pressure for Potential Industrial Applications. *Sep. Purif. Technol.* **2023**, *325*, 124660. https://doi.org/10.1016/j.seppur.2023.124660.
7. Lässig, D.; Lincke, J.; Moellmer, J.; Reichenbach, C.; Moeller, A.; Gläser, R.; Kalies, G.; Cychosz, K. A.; Thommes, M.; Staudt, R.; Krautscheid, H. A Microporous Copper Metal-Organic Framework with High H_2 and CO_2 Adsorption Capacity at Ambient Pressure. *Angew. Chem. Int. Ed.* **2011**, *50* (44), 10344–10348. https://doi.org/10.1002/anie.201102329.
8. Oktavian, R.; Goeminne, R.; Glasby, L. T.; Song, P.; Huynh, R.; Taheri Qazvini, O.; Ghaffari-Nik, O.; Masoumifard, N.; Cordiner, J. L.; Hovington, P.; Van Speybroeck, V.; Moghadam, P. Z. Gas Adsorption and Framework Flexibility of CALF-20 Explored via Experiments and Simulations. *Nat. Commun.* **2024**, *15* (3898). https://doi.org/10.1038/s41467-024-48136-0.
9. Brandenburg, K.; Putz, H. Diamond Version 3.2k, Bonn, **2014**
10. J. Lincke, D. Lässig, M. Kobalz, J. Bergmann, M. Handke, J. Möllmer, M. Lange, C. Roth, A. Moeller, R. Staudt, H. Krautscheid, An Isomorphous Series of Cubic, Copper Based Triazolyl Isophthalate MOFs: Linker Substitution and Adsorption Properties, *Inorg. Chem.* **2012**, *51*, 7579-7586.

11. NETZSCH-Gerätebau GmbH. Proteus Analysis; NETZSCH-Gerätebau GmbH: Selb, Germany, **2009**.
12. Fairley, N.; Fernandez, V.; Richard-Plouet, M.; Guillot-Deudon, C.; Walton, J.; Smith, E.; Flahaut, D.; Greiner, M.; Biesinger, M.; Tougaard, S.; Morgan, D.; Baltrusaitis, J. Systematic and Collaborative Approach to Problem Solving Using X-Ray Photoelectron Spectroscopy. *Appl. Surf. Sci. Adv.* **2021**, *5*, 100112. https://doi.org/10.1016/j.apsadv.2021.100112.
13. Roy, K.; Gopinath, C. S. UV Photoelectron Spectroscopy at near Ambient Pressures: Mapping Valence Band Electronic Structure Changes from Cu to CuO. *Anal. Chem.* **2014**, *86* (8), 3683– 3687, DOI: 10.1021/ac4041026
14. Stoll, S.; Schweiger, A. EasySpin, a Comprehensive Software Package for Spectral Simulation and Analysis in EPR. *J. Magn. Reson.* **2006**, *178* (1), 42–55. https://doi.org/10.1016/j.jmr.2005.08.013.
15. Bleaney, F.R.S; Bowers, K.D. Anomalous Paramagnetism of Copper Acetate. *Proc. R. Soc. Lond. A. Math. Phys. Sci.* **1952**, *214 (1119)*, 451–465. https://doi.org/10.1098/RSPA.1952.0181.
16. Panella, B.; Hirscher, M. Hydrogen Physisorption in Metal–Organic Porous Crystals. *Adv. Mater.* **2005**, *17* (5), 538–541. https://doi.org/10.1002/adma.200400946.
17. Han, D.; Jiang, F.-L.; Wu, M.-Y.; Chen, L.; Chen, Q.-H.; Hong, M.-C. A Non-Interpenetrated Porous Metal–Organic Framework with High Gas-Uptake Capacity. *Chem. Commun.* **2011**, *47* (35), 9861. https://doi.org/10.1039/c1cc12858b.
18. Wen, L.; Shi, W.; Chen, X.; Li, H.; Cheng, P. A Porous Metal-Organic Framework Based on Triazoledicarboxylate Ligands – Synthesis, Structure, and Gas-Sorption Studies. *Eur. J. Inorg. Chem.* **2012**, *2012* (22), 3562–3568. https://doi.org/10.1002/ejic.201200127.
19. Chen, Z.; Xiang, S.; Arman, H. D.; Li, P.; Tidrow, S.; Zhao, D.; Chen, B. A Microporous Metal – Organic Framework with Immobilized –OH Functional Groups within the Pore Surfaces for Selective Gas Sorption. *Eur. J. Inorg. Chem.* **2010**, *2010* (24), 3745–3749. https://doi.org/10.1002/ejic.201000349.
20. Schmitz, B.; Krkljus, I.; Leung, E.; Höffken, H. W.; Müller, U.; Hirscher, M. A High Heat of Adsorption for Hydrogen in Magnesium Formate. *ChemSusChem* **2010**, *3* (6), 758–761. https://doi.org/10.1002/cssc.200900290.
21. He, Y.; Zhang, Z.; Xiang, S.; Fronczek, F. R.; Krishna, R.; Chen, B. A Microporous Metal–Organic Framework for Highly Selective Separation of Acetylene, Ethylene, and Ethane from Methane at Room Temperature. *Chem. Eur. J.* **2012**, *18* (2), 613–619. https://doi.org/10.1002/chem.201102734.
22. Das, M. C.; Xu, H.; Xiang, S.; Zhang, Z.; Arman, H. D.; Qian, G.; Chen, B. A New Approach to Construct a Doubly Interpenetrated Microporous Metal–Organic Framework of Primitive Cubic Net for Highly Selective Sorption of Small Hydrocarbon Molecules. *Chem. Eur. J.* **2011**, *17* (28), 7817–7822. https://doi.org/10.1002/chem.201100350.

23. Chen, B.; Liang, C.; Yang, J.; Contreras, D. S.; Clancy, Y. L.; Lobkovsky, E. B.; Yaghi, O. M.; Dai, S. A Microporous Metal–Organic Framework for Gas-Chromatographic Separation of Alkanes. *Angew. Chem. Int. Ed.* **2006**, *45* (9), 1390–1393. https://doi.org/10.1002/anie.200502844.
24. Muhammad, R.; Jee, S.; Jung, M.; Park, J.; Kang, S. G.; Choi, K. M.; Oh, H. Exploiting the Specific Isotope-Selective Adsorption of Metal–Organic Framework for Hydrogen Isotope Separation. *J. Am. Chem. Soc.* **2021**, *143* (22), 8232–8236. https://doi.org/10.1021/jacs.1c01694.
25. Yushin, G.; Dash, R.; Jagiello, J.; Fischer, J. E.; Gogotsi, Y. Carbide-Derived Carbons: Effect of Pore Size on Hydrogen Uptake and Heat of Adsorption. *Adv. Funct. Mater.* **2006**, *16* (17), 2288–2293. https://doi.org/10.1002/adfm.200500830.
26. Nuhnen, A.; Janiak, C. A Practical Guide to Calculate the Isosteric Heat/Enthalpy of Adsorption: Via Adsorption Isotherms in Metal-Organic Frameworks, MOFs. *Dalton Trans.* **2020**, *49*, 10295–10307. https://doi.org/10.1039/d0dt01784a.
27. Oh, H.; Hirscher, M. Quantum Sieving for Separation of Hydrogen Isotopes Using MOFs. *Eur. J. Inorg. Chem.* **2016**, *2016* (30), 4278–4289. https://doi.org/10.1002/ejic.201600253.
